# Stabilities of Three Key Biological Trisulfides with Implications for Their Roles in the Release of Hydrogen Sulfide and Bioaccumulation of Sulfane Sulfur

**DOI:** 10.1021/acsomega.2c00736

**Published:** 2022-03-22

**Authors:** Eric M. Brown, Ned B. Bowden

**Affiliations:** Department of Chemistry, University of Iowa, Iowa City, Iowa 52242, United States

## Abstract

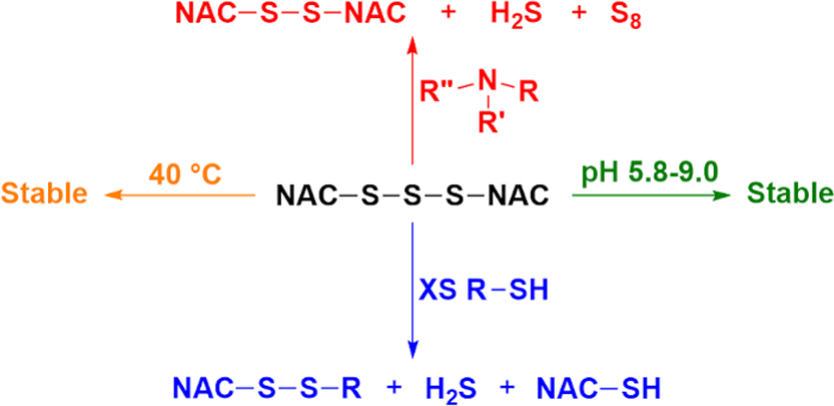

Trisulfides and higher polysulfides are important in the body due to their function as key reservoirs of sulfane sulfur and their rapid reactions to release persulfides. Recent work has shown that persulfides act as powerful antioxidants and release hydrogen sulfide, an emerging gasotransmitter with numerous therapeutic effects. Despite the important role of polysulfides, there is a lack of understanding of their stabilities in aqueous systems. To investigate the reactivity of trisulfides and polysulfides, three key biologically important trisulfides were synthesized from cysteine, glutathione, and *N*-acetylcysteine, and the tetrasulfide of *N*-acetylcysteine was synthesized as a representative polysulfide. The stabilities of sulfides were monitored in buffered D_2_O using ^1^H NMR spectroscopy under a range of conditions including high temperatures and acidic and alkaline environments. The tri- and tetrasulfides degraded rapidly in the presence of primary and tertiary amines to the corresponding disulfide and elemental sulfur. The half-lives of *N*-acetylcysteine tri- and tetrasulfides in the presence of butylamine were 53 and 1.5 min, respectively. These results were important because they suggest that tri- and tetrasulfide linkages are short-lived species *in vivo* due to the abundance of amines in the body. Under basic conditions, cysteine and glutathione trisulfides were unstable due to the deprotonation of the ammonium group, exposing an amine; however, *N*-acetylcysteine trisulfide was stable at all pH values tested. Hydrogen sulfide release of each polysulfide in the presence of cysteine was quantified using a hydrogen sulfide-sensitive electrode and ^1^H NMR spectroscopy.

## 1 Introduction

**Figure 1:**
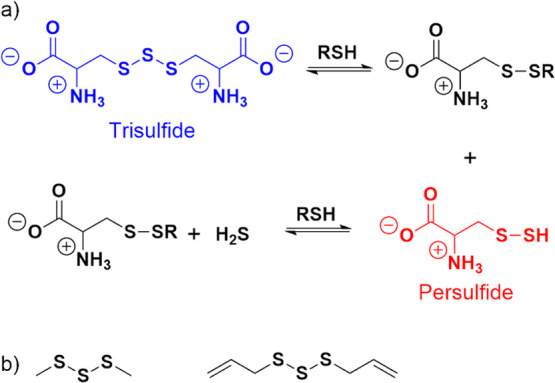
(a) Reaction of cysteine trisulfide and thiols to form persulfide and disulfide. (b) Structures of dimethyl trisulfide (left) and diallyl trisulfide (right).

Trisulfides of cysteine and glutathione are two of the key reservoirs of sulfane sulfur (sulfane sulfur has an oxidation state of zero) that were recently recognized as critical forms of sulfur in the human body.^1−3^ Trisulfides are in rapid equilibrium with thiols to produce reactive persulfides (RSSH) and hydrogen sulfide (H_2_S) as shown in Figure 1a.^2−5^ Although H_2_S is well known as a poisonous gas, it is critically important for human health because it is the third gasotransmitter in human cells along with carbon monoxide and nitric oxide.^6−9^ It is produced by enzymes in the body and used to affect numerous enzymatic cycles.^10−12^ It is emerging as an important chemical that has been implicated in a wide range of diseases and cancers such as prostrate, breast, ovarian, melanoma, and more.^12−23^ The levels of H_2_S *in vivo* are carefully controlled at nanomolar concentrations in cells due to its negative effects at micromolar and higher concentrations,^24^ and it is believed that trisulfides of cysteine and glutathione are two important reservoirs of H_2_S in biological systems.^1−3^ Trisulfides are also found as key structural bonds in proteins between two cysteine groups.^25−27^

When trisulfides react with thiols, they yield persulfides^2−5^ that are stronger nucleophiles than thiols, which makes them more reactive toward toxic electrophiles in cells and better scavengers of reactive oxygen species.^5^ Cysteine persulfide (CysSSH) has been shown to aid in the regulation of insulin secretion, tRNA methythiolation, and Ca^2+^ signaling by Ca^2+^/calmodulin-dependent kinase I, as strong antioxidants to regulate the levels of reactive oxygen species, and as chemicals that promote anti-inflammatory processes.^2,28−31^ It has been hypothesized that persulfides are key chemicals that have wide biological effects *in vivo* rather than H_2_S. Although the roles and relative importance of persulfides and H_2_S remain to be determined, one important route for their formation is the reaction of trisulfides with thiols (Figure 1a). Recent work has strongly suggested that polysulfides (*S* ≥ 3) are present at μM levels in human plasma and that higher concentrations are likely to be incorrect.^2,32^ Little work has been done on the stabilities of tri- and polysulfides that could offer insight into which value is more accurate. In this paper, we report the synthesis of trisulfides from cysteine, *N*-acetylcysteine, and glutathione as well as the tetrasulfide of *N*-acetylcysteine as an example of a polysulfide. The stabilities of these chemicals were investigated in water at different pH levels and in the presence of additives. These studies aid in the interpretation of the importance of trisulfides *in vivo* by quantification of their stabilities in aqueous media.

Most studies to investigate the stabilities of trisulfides have focused on the stability of diallyl trisulfide (DATS) (Figure 1b), which is a component of garlic oil. This trisulfide has been investigated for numerous possible applications including as an anticancer agent, as a source of H_2_S, and much more.^33−35^ Although the medicinal properties of organotrisulfides such as DATS have been extensively researched, their stabilities under a variety of conditions have not. When isolated as a pure liquid, DATS was stable, but the stability decreased when heated. Experiments at room temperature and 35 °C showed that 11 and 30% of neat DATS degraded after 3 months, respectively, but at 4 °C, neat DATS was stable and no change was observed by HPLC after 3 months.^36^ In the presence of glutathione (GSH), DATS degraded rapidly and released H_2_S instantaneously. GSH reacted with DATS in a thiol-disulfide exchange reaction, leaving oxidized GSH as one of the degradation products along with H_2_S and others.^35^ DATS when dissolved in a micellar solution composed of propylene glycol, ethanol, Tween 80, and water was shown to be stable at pH values ranging from 2.6 to 7.0 for 15 min as measured by HPLC. In the same micellar solution, it was found that DATS was not stable in the presence of antioxidants.^37^ The stability of dimethyl trisulfide (Figure 1b) has also been investigated due to its importance as an antidote to cyanide poisoning.^38^ In one study, the stability of neat dimethyl trisulfide (DMTS) was tested at 4, 22, and 34 °C over 1 year. At 4 and 22 °C, DMTS showed no degradation throughout the study. When heated to 34 °C, 70% of DMTS degraded and the degradation products included dimethyl disulfide, dimethyl tetrasulfide, and dimethyl pentasulfide.^39^ In another study, a mixture of neat dimethyl disulfide (DMDS) and dimethyl polysulfides was heated to 150 °C for 48 h and they degraded to methanethiol, carbon disulfide, and H_2_S.^40^

Despite the importance of trisulfides in human health, their stabilities have been sparingly investigated in water and little has been reported on the stabilities of trisulfides based on cysteine, *N*-acetylcysteine (NAC), and glutathione, although these are the most important trisulfides in nature and may provide insights into the stabilities of trisulfides in proteins. To understand how tri- and polysulfides behave in the body, we investigated the stability of the biologically relevant trisulfides of cysteine, glutathione, and *N*-acetylcysteine, along with *N*-acetylcysteine tetrasulfide. We subjected the tri- and tetrasulfides to various conditions and tracked their degradation via ^1^H NMR spectroscopy. We then quantified the H_2_S release of the trisulfides in the presence of cysteine using a H_2_S sensor and obtained the estimated rate constant for each reaction. This paper will report some of the key aspects of the stabilities of these sulfides in buffered water to aid in the interpretation of their possible roles in biological systems.

## 2 Results and Discussion

### 2.1 Synthesis of Tri- and Tetrasulfides

**Figure 2:**
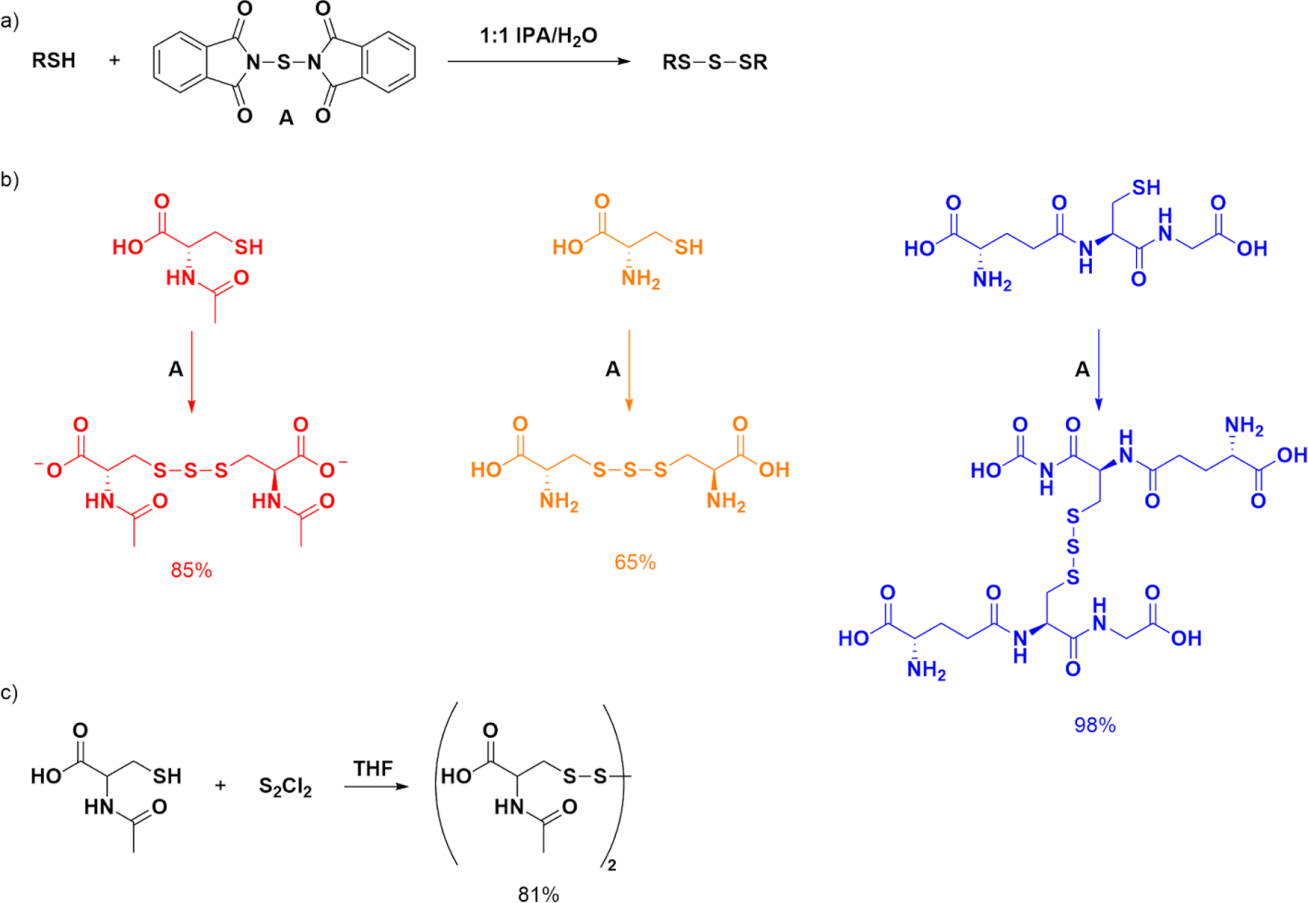
(a) General reaction for the formation of trisulfides. (b) Synthesis of NAC trisulfide (left), cysteine trisulfide (middle), and glutathione trisulfide (right). (c) The synthesis of NAC tetrasulfide is shown.

The synthesis of trisulfides from NAC, cysteine, and glutathione is shown in Figure 2b. Each thiol was reacted with monosulfide transfer reagent **A** in a 1:1 isopropanol:water mixture and isolated in yields between 65 and 85%. The trisulfides of NAC and glutathione were isolated as pure solids, but cysteine trisulfide was unable to be isolated as a single component; it was isolated in a trisulfide:disulfide ratio of 65:35. Numerous reactions were completed to increase the yield of cysteine trisulfide, but it could not be synthesized in higher purity. A key reason for the difficulty in synthesizing cysteine trisulfide was that cysteine was more reactive than NAC and glutathione, which allowed cysteine to react with the trisulfide as it was being formed and degrade the trisulfide into disulfide. The synthesis of NAC tetrasulfide (Figure 2c) was known^41^ and used S_2_Cl_2_ as the disulfide transfer reagent.

### 2.2 Stability of *N*-Acetylcysteine Trisulfide

**Figure 3:**
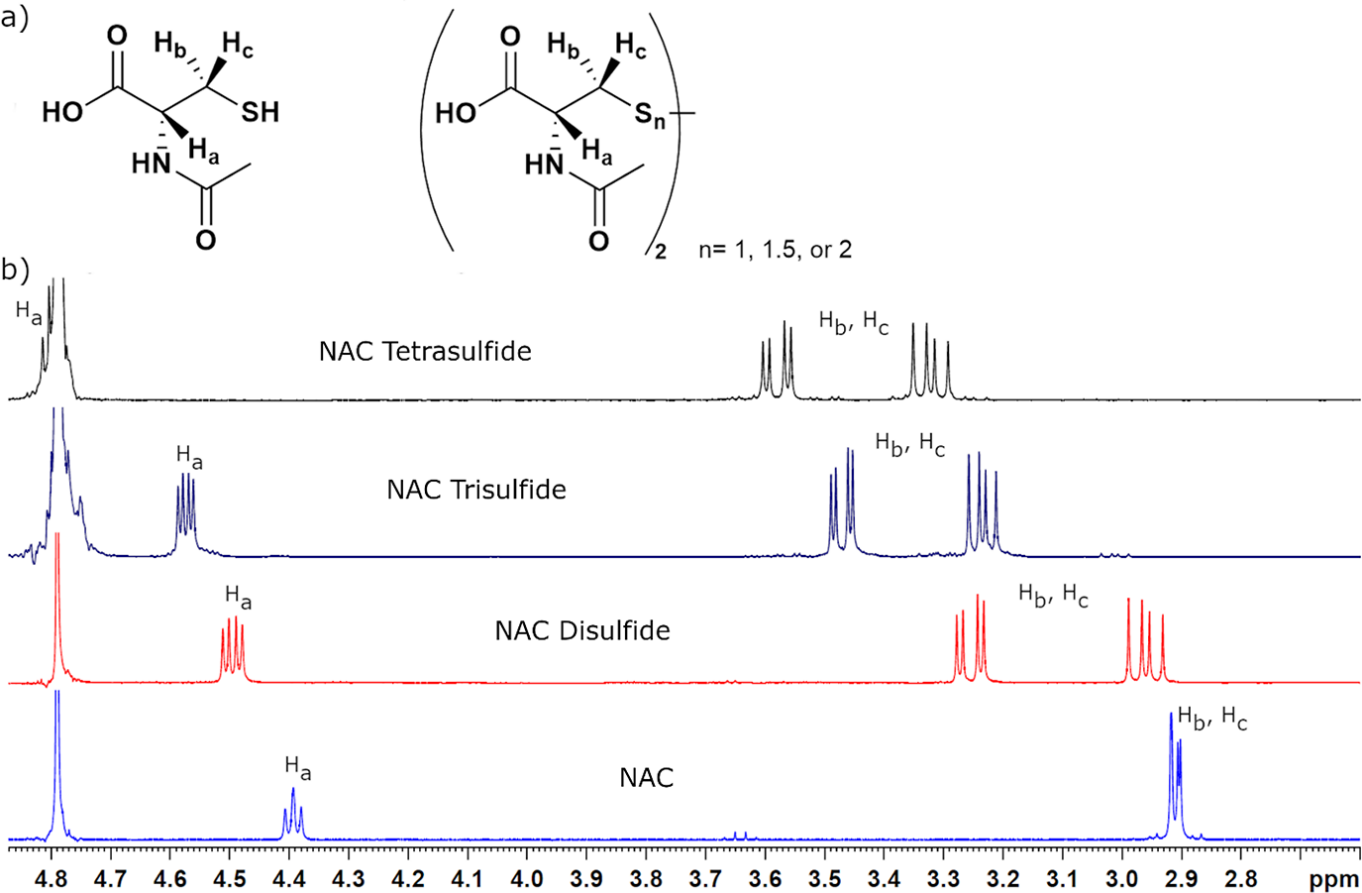
(a) The general structure of NAC polysulfides is shown. (b) Relevant regions of the ^1^H NMR spectra of NAC (bottom), NAC disulfide (red), NAC trisulfide (purple), and NAC tetrasulfide (black) are shown. The peak for HOD at 4.8 ppm partially obscured the peak from Hd of the tetrasulfide.

The ^1^H NMR spectra of NAC and the disulfide, trisulfide, and tetrasulfide of NAC had peaks at unique values for chemical shifts and allowed for their easy characterization (Figure 3). Pure chemicals of each sulfide were synthesized and characterized by ^1^H and ^13^C NMR spectroscopy as well as by high-resolution mass spectroscopy. The unique chemical shifts of the peaks for the Ha and Hb/Hc protons on the sulfides allowed the integration of each sulfide to be determined.

The stability of NAC trisulfide in water buffered at pH values of 5.8, 7.4, and 9.0 was measured. At each pH value, the NAC trisulfide was stable with no observable degradation after 8 days as shown in Figure S6. This result was important because it demonstrated that the trisulfide was stable in water for extended periods of time despite the presence of acids and amides. The stability of NAC trisulfide was investigated in methanol-*d*_4_ and no observable degradation was observed after 8 days. The trisulfide had very limited solubility in CDCl_3_ and acetone, so its stability could not be investigated in these solvents.

**Figure 4:**
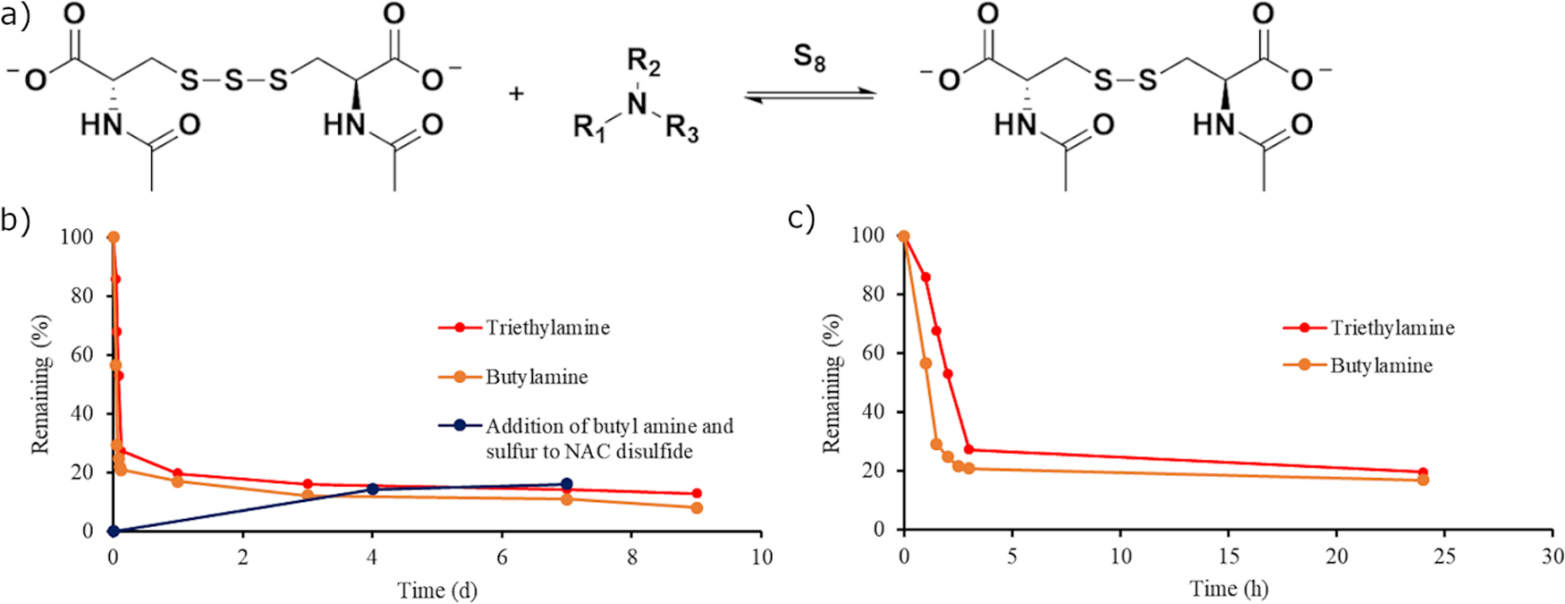
(a) General scheme of the equilibrium between NAC tri- and disulfides in the presence of an amine and elemental sulfur. (b) Stability of NAC trisulfide in the presence of butylamine (orange) and triethylamine (red) measured by ^1^H NMR spectroscopy. In a separate experiment, butylamine and sulfur were added to an aqueous solution of NAC disulfide to investigate the equilibrium between di- and trisulfides whose concentration was shown by the blue line. (c) Early data points of the addition of butylamine (orange) and triethylamine (red) to NAC trisulfide to illustrate that the equilibrium was reached in less than 5 h.

The stability of NAC trisulfide in the presence of a primary or tertiary amine was investigated with the addition of 1 equiv of butylamine or triethylamine to NAC trisulfide in D_2_O in a capped NMR tube (Figure 4). The NAC trisulfide degraded rapidly with 43% (butylamine) and 14% (triethylamine) degraded after 1 h and 92 and 87% after 24 h. The degradation products were primarily the disulfide and an off-white solid, likely elemental sulfur. Only very small amounts of tetrasulfide (<3%) were observed in both reactions. To determine if H_2_S was a byproduct in the reaction, a lead acetate strip was sealed in a vial with NAC trisulfide and butylamine. The lead acetate strip darkened, confirming that H_2_S was released in the reaction. To investigate if nitriles or amides degraded NAC trisulfide, acetonitrile and benzamide were added to NAC trisulfide dissolved in D_2_O. No degradation was observed after 8 days.

To investigate if the insoluble solid was elemental sulfur, the solid was isolated by vacuum filtration and washed with water. The solid did not show any peaks in the ^1^H or ^13^C NMR spectra, so it was reacted with triphenyl phosphine. Triphenyl phosphine is well known to react with elemental sulfur to yield triphenyl phosphine sulfide.^42^ After 16 h of stirring, the ^31^P NMR spectrum showed that triphenyl phosphine sulfide had formed, confirming that the insoluble material was elemental sulfur (Figure S7).

The degradation of NAC trisulfide in the presence of amines leveled off as it reached equilibrium between the di-, tri-, and tetrasulfides as shown in Figure 4. We hypothesized that this was due to the equilibrium between these sulfides in the presence of sulfur and an amine. To test this hypothesis, a reaction was completed in D_2_O with elemental sulfur, NAC disulfide, and butylamine. After 4 days, 15% of NAC disulfide was converted to trisulfide with a small amount of tetrasulfide present (Figure S8). The ^1^H NMR spectrum after day 7 showed no change from the day 3 ^1^H NMR spectrum, which showed that the reaction had reached equilibrium by day 3 (Figure 4). This reaction had not been reported in prior work, so we investigated it. In related prior work by others, it was shown that equilibrium existed between di-, tri-, and polysulfides and the corresponding thiol in the presence of H_2_S.^2−5,43^ In these prior experiments, no thiol was observed by ^1^H NMR, but lead acetate strips detected H_2_S.

To investigate the reaction between disulfides and trisulfides with amines, NAC trisulfide, butylamine, and *N*-ethylmaleimide (NEM) were dissolved in methanol. NEM can rapidly trap thiols and persulfides, and we wanted to investigate if NAC or NAC persulfide could be trapped and detected. Aliquots of this reaction were periodically removed for analysis by high-resolution mass spectroscopy, but the spectra showed no evidence of a thiol or persulfide. Although a negative result, NEM may have reacted with the amines or the persulfide trapped with NEM may have reacted with the amines. Another attempt to understand the mechanism was done by dissolving NAC trisulfide, cysteine trisulfide, and butylamine in water. We hypothesized that if the unsymmetrical disulfide between NAC and cysteine was observed, a thiol or persulfide must be generated. Mass spectrometry was used, and the spectrum showed the mass corresponding to the mixed disulfide (Figure S9). These results indicate a complex mechanism between trisulfides and amines.

To our knowledge, the equilibrium between di- and polysulfides in the presence of an amine and elemental sulfur has yet to be reported. In 1958, Minoura discovered that benzyl tri- and tetrasulfides and *p*-tolyl tri- and tetrasulfides degraded to the corresponding disulfide in the presence of an amine.^44^ The author did not report the equilibrium between the sulfides. A recent report showed that aminothiol compounds help solubilize elemental sulfur in water as more reactive polysulfides, which were stabilized by hydrogen bonding with the ammonium.^45^ The reaction between thiols and sulfur is well known to produce trisulfides and polysulfides and amines have been reported to catalyze this reaction, but these reactions required the addition of thiols and sulfur often in large excess.^46−49^ In contrast, we report the first reaction of a disulfide in the absence of thiols that reacts with elemental sulfur and catalyzed by amines to produce trisulfides.

Estimated rate constants and half-lives were obtained for the degradation of NAC trisulfide in the presence of butylamine and triethylamine. Due to the equilibrium at extended times, only the data points up to 2 h were used. The graphs are shown in Figure S10, and the estimated half-lives for the degradation of NAC trisulfide in the presence of butylamine and triethylamine were found to be 0.94 and 2.2 h, respectively. This data showed that amines degraded trisulfides rapidly and primary amines degraded the trisulfide faster than tertiary amines, which is the same observation described in the literature.^44^ This result is important for the biological role of trisulfides since amines are present as small chemicals and within proteins and they are likely to degrade trisulfides. As an example, the amino acid l-lysine has an average plasma concentration of 275.8 μmol/L.^50^

Because zinc and iron are important metals found throughout the human body and both metals form stable metal sulfides, the stability of NAC trisulfide to these metals was investigated.^51,52^ Zinc sulfate and iron sulfate were added to a solution of NAC trisulfide dissolved in D_2_O. Surprisingly, the trisulfide was stable for 11 days in the presence of both metals, with no precipitate or observable degradation by NMR spectroscopy.

All the stability studies were completed at room temperature, so further studies were completed to investigate the thermal stability of NAC trisulfide. To test the stability of NAC trisulfide at elevated temperatures, an NMR tube of the trisulfide in water was stored in a 40 °C oil bath, and the ^1^H NMR spectra were periodically obtained. No noticeable degradation was observed after 10 days. When the temperature was increased to 60 °C, 74% of NAC trisulfide degraded in 3 days with the degradation products as the corresponding disulfide and sulfur. This data demonstrates that NAC trisulfide degrades in the presence of heat but is stable at body temperature.

### 2.3 Stability of Cysteine Trisulfide

The ^1^H NMR spectra of cysteine and the di- and trisulfides of cysteine are shown in Figure S11. The synthesis of the trisulfide was challenging and only yielded cysteine trisulfide at a purity of 65% with the remainder as disulfide and a small amount of tetrasulfide. Fortunately, the peaks for cysteine and di-, tri-, and tetrasulfides were well resolved from each other and allowed their concentrations to be measured independently.

The stability of cysteine trisulfide was investigated in buffered water at pH values of 5.8, 7.0, and 9.0. A pH of 7.4 was attempted due to its biological importance, but cysteine trisulfide was poorly soluble at this pH and no peaks in the ^1^H NMR spectrum were observed. At pH values of 5.8, 7.0, and 9.0, the cysteine trisulfide was soluble and its ^1^H NMR spectra were obtained.

At an acidic pH of 5.8, cysteine trisulfide was stable with <3% degradation over 9 days (Figure S12). When cysteine trisulfide was added to water buffered at a neutral pH of 7.0, 40% of the cysteine trisulfide degraded after 13 days with the degradation products of cystine (cystine is a common name for cysteine disulfide) and an insoluble solid, which was identified as elemental sulfur by reaction with triphenylphosphine. When the pH was increased to 9.0, cysteine trisulfide was 56% degraded after 13 days with the degradation products of cystine and elemental sulfur. Rate constants and half-lives were obtained for the degradation of cysteine trisulfide at pH 7.0 and pH 9.0 using the data points up to 13 days. At pH 7.0 and 9.0, the estimated half-lives were 16.9 days (407.7 h) and 11.4 days (277.3 h), respectively (Figure S12). This data shows that cysteine trisulfide is less stable in more alkaline environments. This information is useful in understanding how trisulfides behave in different parts of the body that vary in pH including tumor cells (pH 6.4–7.0), healthy tissues (pH 7.2–7.5), and pancreatic fluid (pH ∼8.8).^53,54^

**Figure 5:**
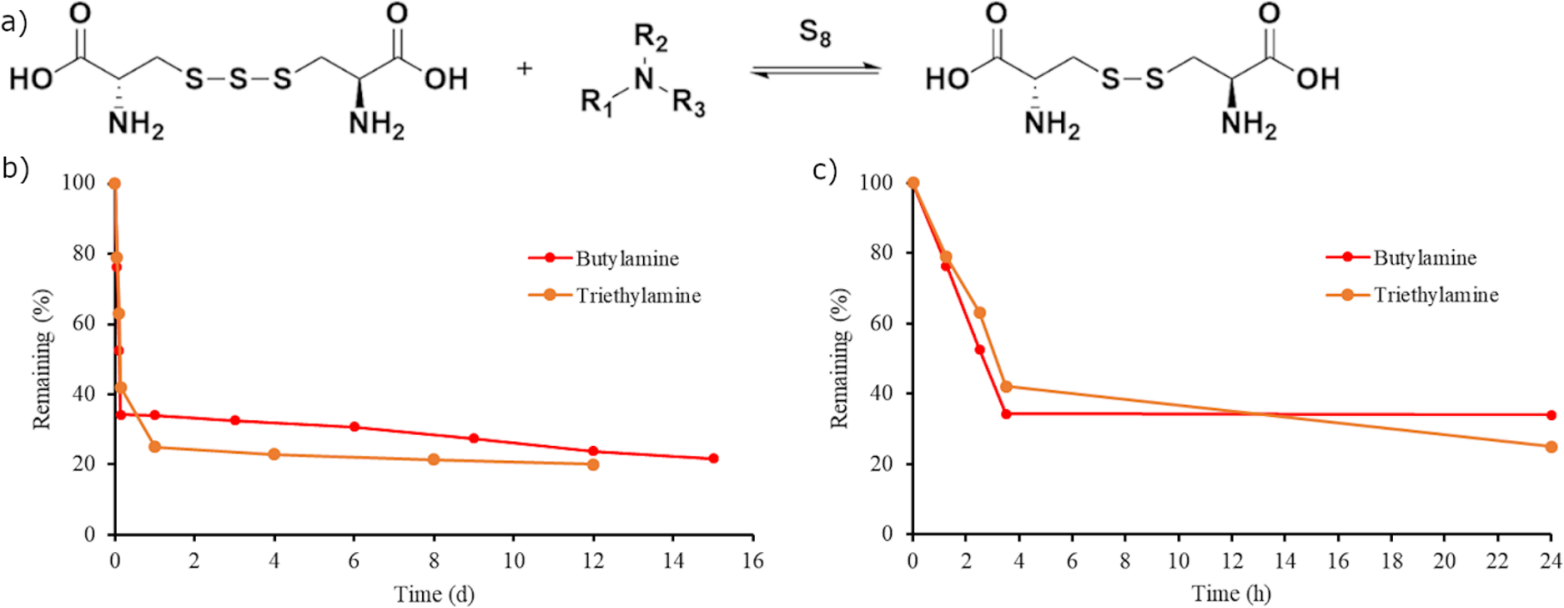
(a) General scheme of the equilibrium between cysteine trisulfide and cystine in the presence of an amine and elemental sulfur. (b) The stability of cysteine trisulfide in the presence of butylamine (red) and triethylamine (orange) measured by ^1^H NMR spectroscopy is shown. (c) Early data points of the degradation of cysteine trisulfide in the presence of butylamine (red) and triethylamine (orange).

Prior experiments that demonstrated that NAC trisulfide was stable in D_2_O at a variety of different pH values but that it was unstable in the presence of amines indicated that the instability of cysteine trisulfide was likely due to the ammonium (p*K*_a_ = 10.8) group that was readily deprotonated at neutral or basic pH values to expose an amine. As the pH of the buffer was increased, the rate of degradation of cysteine trisulfide increased as expected based on the increasing proportion of the ammonium that was deprotonated to yield a neutral, primary amine. This neutral amine can degrade cysteine trisulfide through intra- or intermolecular reactions. This hypothesis was confirmed by the addition of an amine to a solution of cysteine trisulfide. Two experiments were completed using either butylamine or triethylamine in D_2_O with cysteine trisulfide (Figure 5). After 1 day, cysteine trisulfide degraded by 63 and 78% in the presence of 2 equiv of butylamine and triethylamine, respectively, which were faster than the degradation in the absence of added amine. In both reactions with butylamine and triethylamine, the observable degradation products were cystine and elemental sulfur. The degradation of cysteine trisulfide in D_2_O with amines leveled off, reaching a concentration of approximately 20% cysteine trisulfide after 10 days in the presence of butylamine and triethylamine. The cysteine trisulfide did not fully degrade due to the equilibrium between trisulfide and cystine catalyzed by the presence of an amine and elemental sulfur. Estimated half-lives for the degradation of cysteine trisulfide in the presence of butylamine and triethylamine were obtained using early data points unaffected by the equilibrium and found to be 2.3 and 2.9 h, respectively (Figure S13).

The stability of cysteine trisulfide was further investigated by tracking the ^1^H NMR spectrum over time in the presence of acetonitrile and benzamide in D_2_O. A 3:1 mole ratio of either acetonitrile or benzamide to cysteine trisulfide was used to provide an excess of these chemicals. Cysteine trisulfide was stable around both acetonitrile and benzamide, displaying no observable degradation by NMR spectroscopy after 12 days.

### 2.4 Stability of Glutathione Trisulfide

The stability of glutathione trisulfide was investigated at pH values of 5.8, 7.4, and 9.0 (Figure S14a). The degradation of glutathione trisulfide was more rapid at pH values of 5.8 and 7.4 than that of the trisulfides of NAC and cysteine. At an acidic pH of 5.8, 71% of glutathione trisulfide degraded after 10 days. At pH values of 7.4 and 9.0, glutathione trisulfide degraded 79 and 81%, respectively, at day 9. Glutathione trisulfide degraded faster than cysteine trisulfide due to the lower p*K*_a_ of the ammonium on glutathione (p*K*_a_ = 9.6) compared to cysteine (p*K*_a_ = 10.8), allowing for a higher concentration of the neutral amine. The degradation of glutathione trisulfide reached an equilibrium of approximately 20% with the disulfide due to the reaction catalyzed by amine with elemental sulfur. Rate constants and half-lives were obtained for the degradation of glutathione trisulfide at each pH value using early data points unaffected by the equilibrium. At pH values of 5.8, 7.4, and 9.0, the estimated half-lives were 6.3 days (151 h), 0.90 days (21.6 h), and 0.79 days (19.0 h), respectively (Figure S14b). This data shows that glutathione trisulfide is more stable in acidic environments, which is consistent with the results from cysteine trisulfide.

**Figure 6:**
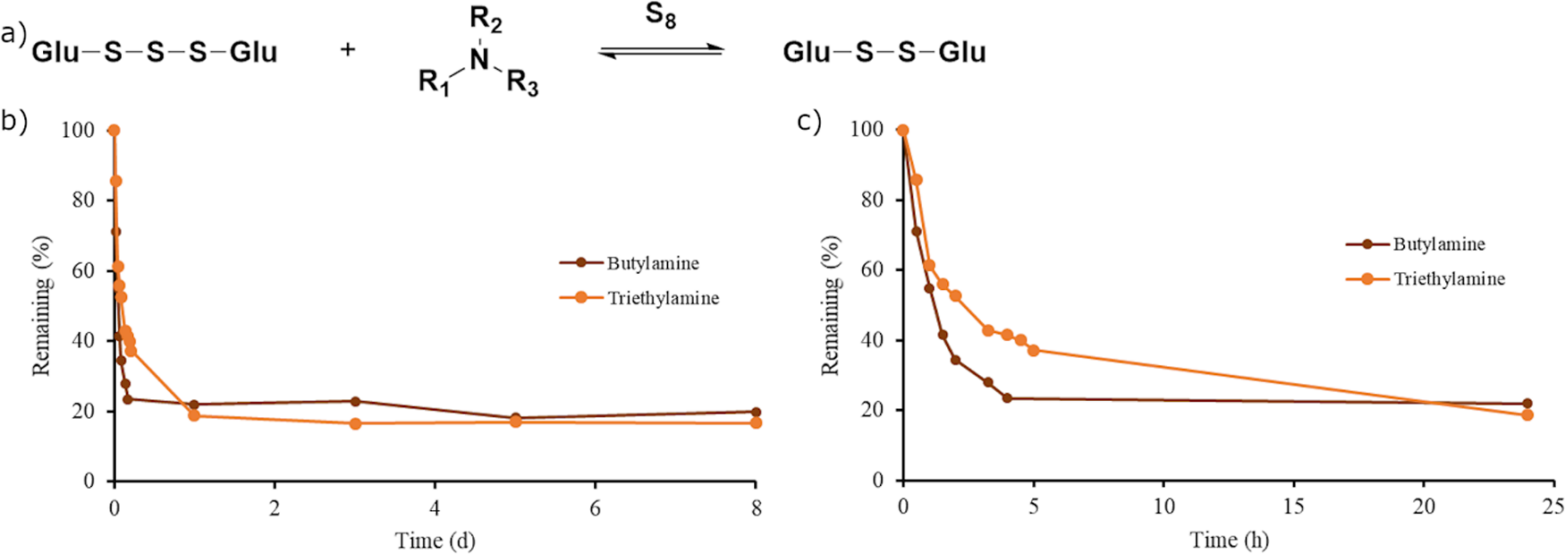
(a) General scheme of the equilibrium between glutathione tri- and disulfides in the presence of an amine and elemental sulfur. (b) The stability of glutathione trisulfide in the presence of butylamine (red) and trimethylamine (orange) measured by ^1^H NMR spectroscopy is shown. (c) Early data points of the degradation of glutathione trisulfide in the presence of butylamine (red) and triethylamine (orange).

The stability of glutathione trisulfide was studied in the presence of butylamine and triethylamine due to the existence of amines in cells (Figure 6). After 30 min, glutathione trisulfide degraded by 29 and 14% in the presence of butylamine and triethylamine, respectively. The degradation products were the corresponding disulfide and elemental sulfur. After 1 day, glutathione trisulfide in the presence of butylamine and triethylamine degraded by 78 and 81%, respectively, and the degradation leveled off due to the equilibrium between disulfide, elemental sulfur, and amine. Estimated half-lives of the degradation of glutathione trisulfide in the presence of butylamine and triethylamine were obtained using early data points unaffected by the equilibrium and found to be 1.3 and 0.72 h, respectively (Figure S15).

### 2.5 Stability of *N*-Acetylcysteine Tetrasulfide

Recent work has shown that tetrasulfides and longer sulfides are found in cells and are sources of sulfane sulfur.^55,56^ The stability of polysulfides longer than trisulfides has not been well studied. One important study showed that organic tetrasulfides, including NAC tetrasulfide, were unstable in the presence of thiols and released H_2_S, although no rate constant was provided.^41^ To investigate the stability of polysulfides, NAC tetrasulfide was synthesized because of the stability of NAC trisulfide in water due to the absence of an ammonium group.

The stability of NAC tetrasulfide was investigated in D_2_O buffered at pH values of 5.8, 7.4, and 9.0 (Figure S16a). At a pH of 5.8, NAC tetrasulfide degraded by 13% after 9 days. At pH values of 7.4 and 9.0, the tetrasulfide degraded by 55 and 52%, respectively, after 9 days. At each pH, the degradation product was NAC trisulfide, elemental sulfur, and a small amount of NAC disulfide (<3%). These results are compared to the absence of any observed degradation of NAC trisulfide in water at these pH values over 8 days. Estimated rate constants and half-lives for the degradation of NAC tetrasulfide at each pH were obtained. At pH values of 5.8, 7.4, and 9.0, the estimated half-lives were 53.3, 7.5, and 8.2 days, respectively (Figure S16b).

**Figure 7:**
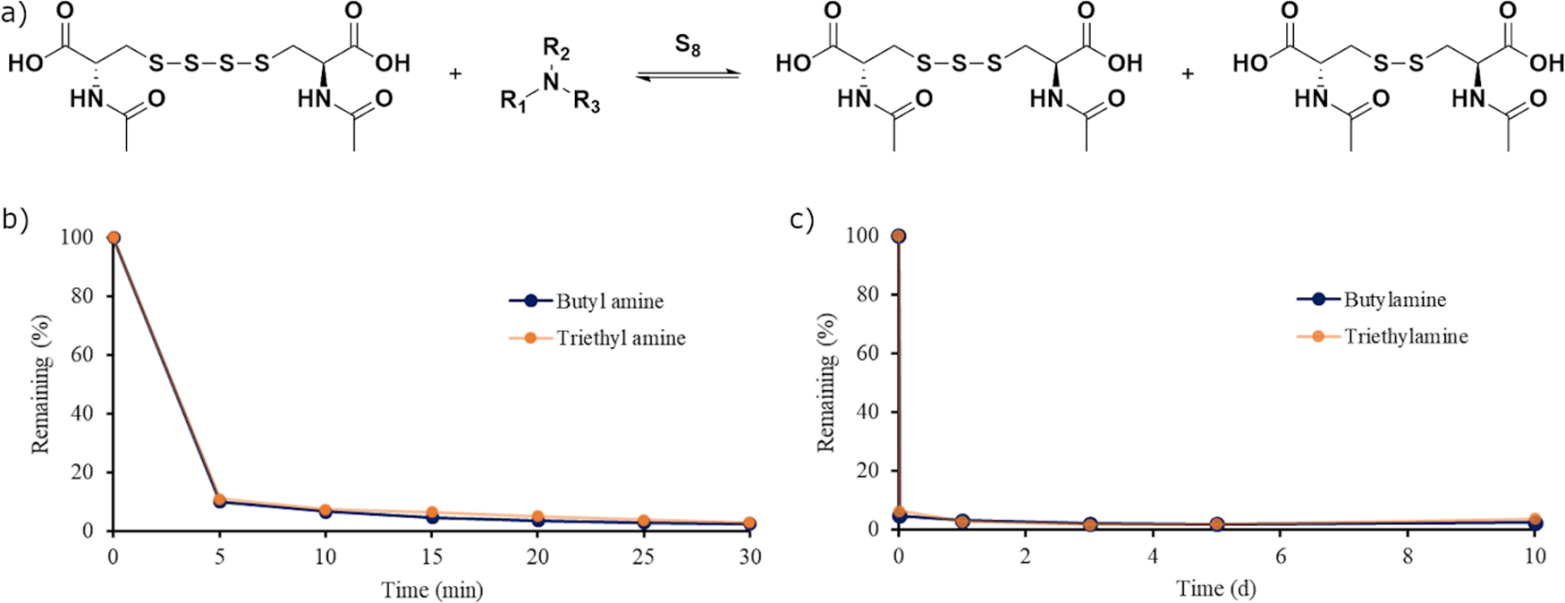
(a) General scheme of the equilibrium between NAC tetra-, tri-, and disulfides in the presence of an amine and elemental sulfur. (b) The stability of NAC tetrasulfide in the presence of butylamine (blue) and triethylamine (orange) measured by ^1^H NMR spectroscopy is shown. (c) The early data points of the reaction between NAC tetrasulfide with butylamine (blue) and triethylamine (orange) are shown.

The stability of NAC tetrasulfide was investigated in the presence of butylamine and triethylamine (Figure 7). The results showed that amines rapidly degraded the tetrasulfide with less than 10% of the tetrasulfide present after 5 min and only 4% of the tetrasulfide present after a day. After 10 days, the observable degradation products were an off-white solid identified as sulfur, NAC trisulfide (18% NAC trisulfide in the reaction with butylamine and 20% NAC trisulfide in the reaction with butylamine), and NAC disulfide (80% NAC disulfide in the reaction with butylamine and 78% NAC disulfide in the reaction with butylamine). As observed in the reaction with NAC trisulfide and amines, the degradation of NAC tetrasulfide with amines leveled off, as shown in Figure 7, due to the equilibrium between polysulfides in the presence of amines and elemental sulfur. An upper limit of the half-life was obtained for the degradation of NAC tetrasulfide in the presence of butylamine and triethylamine due to the equilibrium being reached by 5 min and found to be 1.6 min for both amines (Figure S17). These results show that the degradation of NAC tetrasulfide is very rapid and 2 orders of magnitude faster than that of NAC trisulfide in the presence of amines. Due to the presence of amines existing in living systems, this data suggests that polysulfides (*S* ≥ 4) would be short-lived species *in vivo*.

The degradation of NAC tetrasulfide was tracked in nonbuffered D_2_O at room temperature using ^1^H NMR spectroscopy. After 35 days, the tetrasulfide degraded by 69%. The degradation products were the corresponding trisulfide (63%), a small amount of the corresponding disulfide (∼6%), and an insoluble solid identified as elemental sulfur (Figure S18). An estimation of the kinetics was obtained using the data obtained after 35 days, giving an estimated half-life and rate constant of 17.8 days and 3.9 × 10^–2^ days^–1^, respectively (Figure S19a).

A kinetic study of the degradation of NAC tetrasulfide in nonbuffered D_2_O was completed at 40 °C. The tetrasulfide degraded faster at 40 °C than at room temperature. After 20 days, the tetrasulfide degraded by 83%. An estimated half-life and rate constant were obtained after 20 days and found to be 8.0 days and 8.7 × 10^–2^ days^–1^, respectively (Figure S19b). After 20 days, the observable degradation products were the corresponding trisulfide (∼60%), elemental sulfur, and the corresponding disulfide (∼23%).

Interestingly, the degradation of NAC tetrasulfide at 40 °C leveled off and remained at 83% degradation after 63 days (Figure S20). To understand why the degradation leveled off, NAC trisulfide was dissolved in water, elemental sulfur was added, and the contents were heated to 40 °C. After 15 days, tetrasulfide (11%) and disulfide (12%) had formed in small amounts (Figure S21). Unlike prior reactions reported here, this reaction did not have any amines to catalyze the equilibrium between tri- and tetrasulfides in the presence of sulfur. Prior work reported the reaction of disulfides with elemental sulfur without catalysts to yield polysulfides. For instance, in a report in 2013, diallyl disulfide reacted with elemental sulfur in the absence of a catalyst at 120 °C to yield diallyl polysulfides.^57^ This reaction used sulfur (melting point of 113 °C) as the solvent. In contrast, the reaction investigated here involved the uncatalyzed reaction between NAC trisulfide and NAC tetrasulfide with elemental sulfur in water at a low temperature of 40 °C. Sulfur is only sparingly soluble in water, so it was surprising that it was an effective solvent for this reaction.

### 2.6 Degradation of *N*-Acetylcysteine Trisulfide in the Presence of Butylamine under Biologically Relevant Conditions

**Figure 8:**
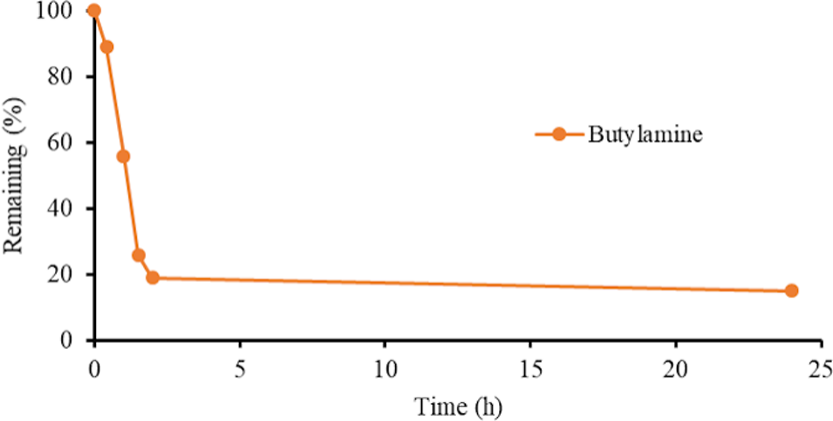
Stability of NAC trisulfide in the presence of butylamine at 37 °C and pH 7.4 measured by ^1^H NMR spectroscopy.

To investigate how amines degrade polysulfides in the body, NAC trisulfide was dissolved in pH 7.4 in D_2_O at a concentration of 10 mM. Although recent work suggests that polysulfides are present at μM concentrations,^2^ these conditions were too dilute to give good resolution in the ^1^H NMR spectrum. Butylamine was added and the reaction was maintained at body temperature (37 °C). Degradation was tracked using ^1^H NMR spectroscopy (Figure 8). NAC trisulfide was used in this experiment due to its stability and solubility at a pH of 7.4.

After 1 h, NAC trisulfide was 45% degraded, and it was 81% degraded after 2 h. The degradation leveled off, remaining at 81% degraded after 1 day due to the equilibrium between the di- and trisulfides in the presence of an amine and elemental sulfur. The observable degradation products were NAC disulfide and elemental sulfur. The estimated half-life was obtained using early data points unaffected by the equilibrium and found to be 0.77 h^–1^ (Figure S22). This result confirms that trisulfides may be short-lived species *in vivo*.

### 2.7 Kinetics of Degradation of Cysteine, Glutathione, and *N*-Acetylcysteine Trisulfide in the Presence of a Thiol

To investigate the rate of reaction of trisulfides with an added thiol and to characterize the degradation products, 5 mM trisulfides of cysteine, glutathione, and NAC were separately added to D_2_O in an NMR tube with 5 equiv of the corresponding thiol and then the NMR tubes were capped. For instance, 5 equiv of cysteine was added to cysteine trisulfide and 5 equiv of NAC was added to NAC trisulfide. The pH was buffered to a pH of 7.0 to ensure that each trisulfide was soluble and to closely mimic *in vivo* pH.

**Figure 9:**
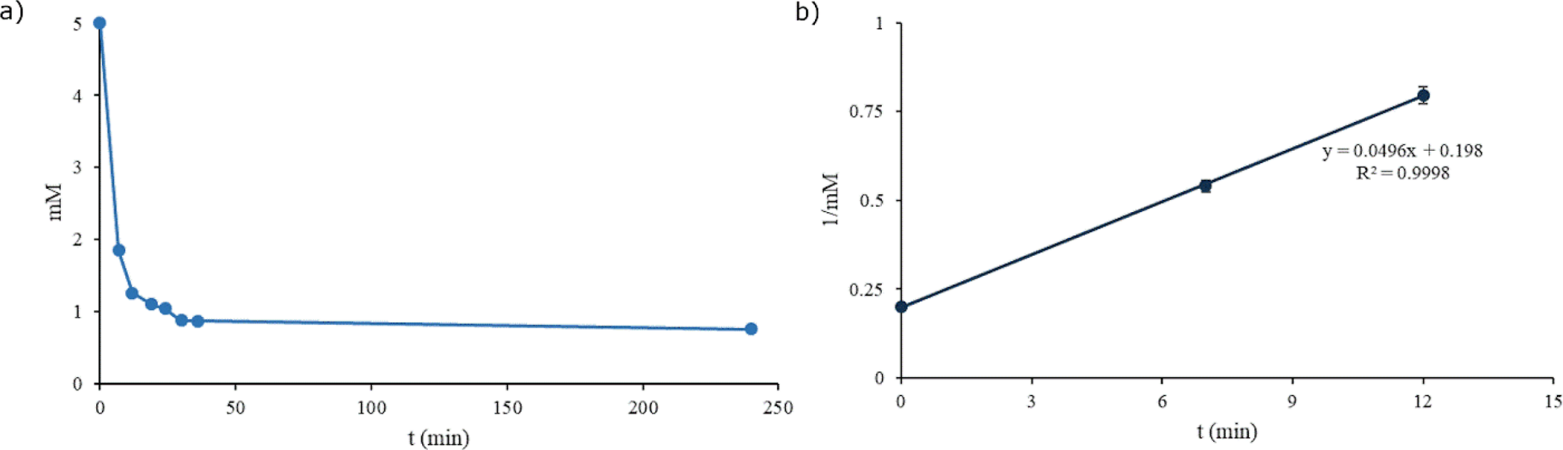
(a) The concentration of NAC trisulfide in the presence of 5 equiv of NAC measured by ^1^H NMR spectroscopy showed equilibrium when in the presence of H_2_S. (b) Estimated kinetics of the degradation of NAC trisulfide in the presence of the corresponding thiol. Error bars are ± instrument error.

The reactions were very rapid; within 7 min, over 60% of the trisulfide was reacted (Figure 9 and Figure S23). For each reaction, the degradation to the corresponding disulfide leveled off and the trisulfide never fully degraded, and a small amount of the tetrasulfide was observed in the ^1^H NMR spectrum. Prior work described the equilibrium between disulfides, trisulfides, polysulfides, and thiols in the presence of H_2_S.^2−5,43^

To investigate whether the presence of H_2_S was the reason for the presence of trisulfides at extended periods of time, two experiments were completed. NAC disulfide was dissolved in water and sodium hydrosulfide was added. After 1 day, NAC trisulfide and NAC were observed by ^1^H NMR spectroscopy, confirming that the equilibrium with aqueous hydrogen sulfide can convert NAC disulfide to NAC trisulfide. To further confirm that H_2_S was important for the equilibrium, NAC trisulfide was dissolved in water and NAC was added. The vial was left uncapped to allow H_2_S to evaporate. After 1 day, no NAC trisulfide was observed by ^1^H NMR spectroscopy, and the only product was the disulfide.

Because of the competing reaction with hydrogen sulfide, an upper limit of the rate constant was obtained for the reaction of trisulfide with the thiol. The reaction of each trisulfide was very rapid and the reactions reached equilibrium within 15 min. With an estimated half-life limit of 4.0 min for each trisulfide, the rate constant of reaction was 4.9 × 10^–2^ M/min (Figure 9 and Figure S23).

The rate of reaction between NAC, cysteine, and glutathione trisulfides with their corresponding thiol (e.g., NAC trisulfide with NAC and glutathione trisulfide with glutathione) was rapid. To investigate whether the thiol was critical for these reactions, we investigated the reaction of each trisulfide with the same thiol. Each trisulfide was separately reacted with NAC, but the peaks in the ^1^H NMR spectra overlapped and were too challenging to interpret. We also investigated 1-propanethiol, 2-mercaptoethanol, and isobutyl mercaptan as examples of primary and secondary thiols, and the reactions of these thiols with the three trisulfides were very rapid and were over 80% complete within 7 min. These reactions demonstrate that the biologically relevant trisulfides react very rapidly with a wide range of thiols.

### 2.8 Measurement of H_2_S Release from Cysteine Trisulfide, Glutathione Trisulfide, *N*-Acetylcysteine Trisulfide, and *N*-Acetylcysteine Tetrasulfide in the Presence of Cysteine

**Figure 10:**
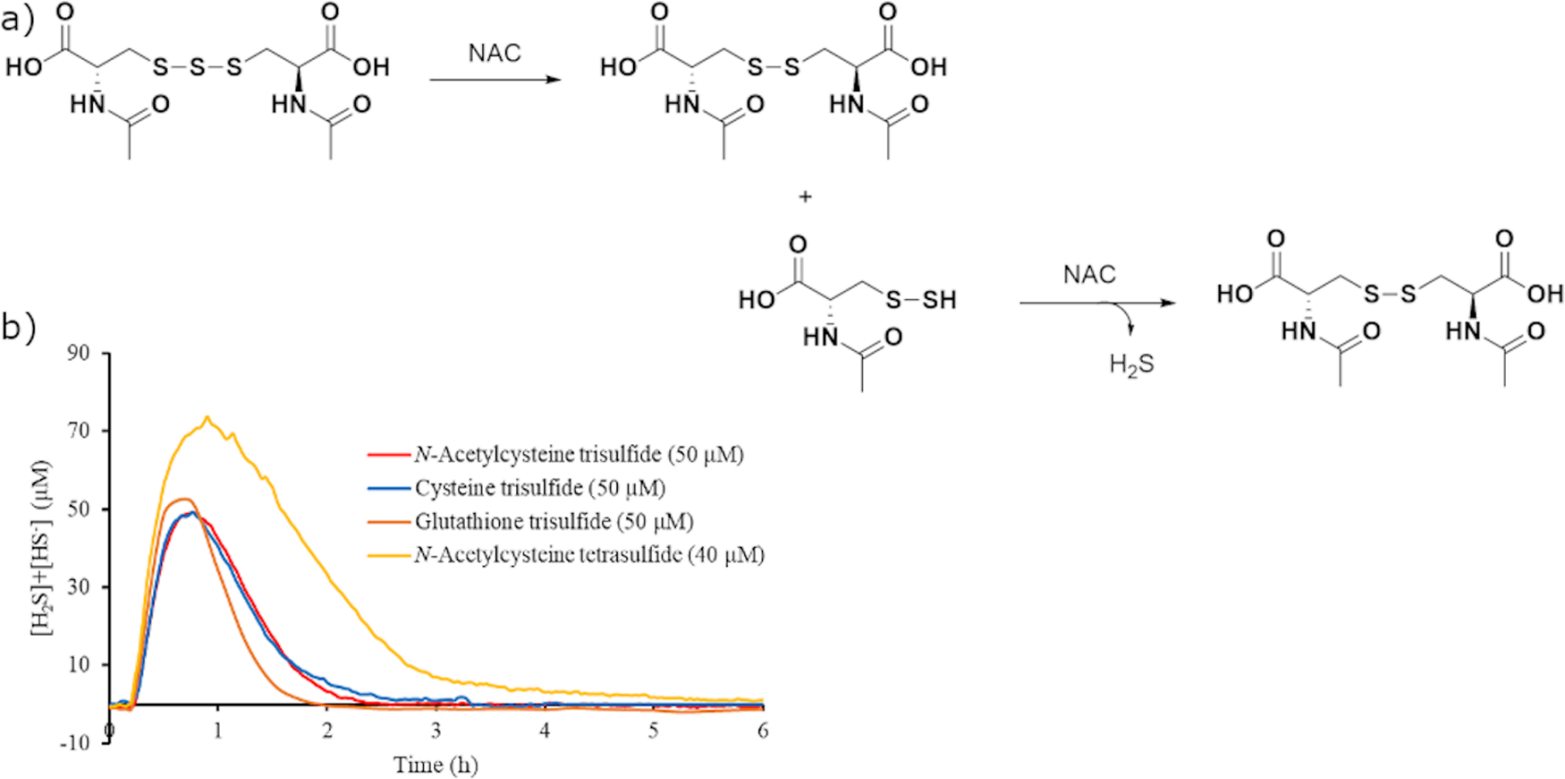
(a) The degradation pathway of NAC trisulfide is shown. (b) The total concentration of H_2_S and HS^–^ from the reaction of cysteine, glutathione, and NAC trisulfide and NAC tetrasulfide in the presence of 10 equiv of cysteine as measured by an H_2_S electrode is shown.

One important role of tri- and polysulfides in cells is to serve as a reservoir for H_2_S.^1−3^ The central sulfur in trisulfides is rapidly reduced upon reaction with two thiols to yield H_2_S (Figure 10a).^2−5^ Tetrasulfides also react rapidly with thiols to generate 2 equiv of H_2_S via the two central sulfurs, although the mechanism is more complex.^41^ To investigate these reactions, the trisulfides of NAC, cysteine, and glutathione and NAC tetrasulfide were reacted with an excess of cysteine in water and the concentration of H_2_S was measured using an H_2_S-sensitive electrode. The electrode allowed the real-time detection of the concentration of H_2_S. Because H_2_S has a p*K*_a_ of 7.0, some of it is present as HS^–^, so a pH electrode was also used to calculate the total concentration of sulfide.

The release of H_2_S from each trisulfide and NAC tetrasulfide was quantified. Each trisulfide was initially dissolved in water buffered at a pH of 6.7 to establish a baseline and then an excess of cysteine (10 equiv) was added. The H_2_S probe monitored the H_2_S release and recorded a reading every 2 s (Figure 10b). The beaker housing this reaction was uncapped so H_2_S evaporated. A 50 μM concentration of each trisulfide was tested. The peak concentration of H_2_S from NAC trisulfide was 49 μM with a peaking time of 46 min. Cysteine trisulfide had a peak H_2_S concentration of 49 μM with a peaking time of 46 min, and glutathione trisulfide had a peaking concentration of 51 μm with a peaking time of 45 min. The initial concentration of NAC tetrasulfide was 40 μM, so the peak H_2_S concentration would not peak above the limit of the H_2_S electrode. The peak H_2_S concentration from NAC tetrasulfide was 74 μM with a peaking time of 56 min. These results show that in the presence of thiols, polysulfides rapidly release H_2_S and that the peak concentration of H_2_S can be nearly equal to the initial concentration of the trisulfide.

## 3 Conclusions

Trisulfides have been hypothesized to be critically important chemicals *in vivo* and to form covalent cross-links within proteins, but their stabilities have not been well studied. This article reports the synthesis of biologically relevant tri- and tetrasulfides and their stabilities under a variety of conditions in aqueous solvents to gain insight into how these chemicals behave in the body. Several important discoveries were made including the finding that amines rapidly degrade tri- and tetrasulfides, suggesting that these polysulfides have limited lifetimes *in vivo*. The degradation of trisulfides in the presence of amines levels off due to the equilibrium between di- and polysulfides in the presence of elemental sulfur and amines. We also showed that at the biological pH of 7.4, trisulfides, such as NAC trisulfide, can be stable if they lack an amine group. Glutathione trisulfide, which mimicked a protein containing a trisulfide linkage in the body, degraded under acidic and alkaline environments and reacted rapidly in the presence of primary and tertiary amines. These results further suggest that trisulfide bonds are very reactive and likely prevalent at μM levels rather than mM concentrations. Although more work needs to be completed to understand the complex equilibria *in vivo* between thiols, persulfides, and trisulfides, this article reports key reactions and half-lives of trisulfides that are expected to be important *in vivo*.

## 4 Experimental Section

### 4.1 Materials and Methods

All chemicals were obtained from Sigma-Aldrich. The NMR spectra were obtained using a Bruker Avance-300 at 300 MHz and a Bruker DRX-400 at 400 MHz. An amperometric H_2_S microsensor for real-time sulfide monitoring was purchased from Analysenmesstechnik GmBH and was calibrated as needed.

### 4.2 2,2′-Thiobis(isoindoline-1,3-dione)

A literature procedure was followed with slight modifications.^58^ To a solution of phthalimide (7.1 g, 48.3 mmol) dissolved in DMF (40 mL), sulfur monochloride (6.4 g, 47.4 mmol) was added. The contents were stirred for 16 h. The white precipitate was collected by vacuum filtration and washed with toluene (10 mL) to give the product in a 75% yield (5.9 g). ^1^H NMR (300 MHz, CDCl_3_) δ 7.88 (m, 4H), 7.77 (m, 4H); ^13^C NMR (75 MHz, CDCl_3_) δ 166.2, 135.2, 131.6, 124.7.

### 4.3 Cysteine Trisulfide

To a solution of 2,2′-thiobis(isoindoline-1,3-dione) (1.0 g, 3.0 mmol) in a 1:1 mixture of water and isopropanol (64 mL), cysteine (0.34 g 2.8 mmol) was added. After stirring for 10 min, the crude product was collected by vacuum filtration and washed with acetone (10 mL) and dichloromethane (10 mL) to give the purified product as a white solid in a 65% yield (265 mg). ^1^H NMR (300 MHz, D_2_O) δ 4.12 (dd, *J* = 8.1, 4.1 Hz, 2H), 3.52 (dd, *J* = 15.1, 4.1 Hz, 2H), 3.31 (dd, *J* = 15.0, 8.2 Hz, 2H); ^13^C NMR (75 MHz, D_2_O) δ 172.7, 53.2, 38.2; ESI-MS: calcd. for C_6_H_12_N_2_O_4_S_3_H^+^, 273.0037; found, 273.0027.

### 4.4 Glutathione Trisulfide

To a solution of glutathione (272 mg, 0.88 mmol) dissolved in a 1:1 mixture of water and isopropanol (14 mL), 2,2′-thiobis(isoindoline-1,3-dione) (322 mg, 0.99 mmol) was added. The corresponding solution was stirred for 15 min. The crude product was collected by vacuum filtration and washed with acetone (5 mL) and dichloromethane (5 mL) to give the purified product as a white solid in a 98% yield (278 mg). ^1^H NMR (300 MHz, D_2_O) δ 3.88 (s, 4H), 3.77 (t, *J* = 6.3 Hz, 2H), 3.42 (dd, *J* = 14.4, 4.7 Hz, 2H), 3.18 (dd, *J* = 14.5, 9.2 Hz, 2H), 2.50 (m, 4H), 2.12 (q, *J* = 7.3 Hz, 4H); ^13^C NMR (75 MHz, D_2_O) δ ESI-MS: calcd. for C_20_H_31_N_6_O_12_S_3_^–^, 643.1162; found, 643.1170.

### 4.5 *N*-Acetylcysteine Trisulfide

A literature procedure was followed with slight modifications.^59^ To a solution of *N*-acetylcysteine (1.0 g, 6.1 mmol) dissolved in a 1:1 mixture of water and isopropanol (20 mL), 2,2′-thiobis(isoindoline-1,3-dione) (2.4 g, 7.4 mmol) was added. The corresponding solution was stirred for 6 h. Sodium bicarbonate (0.51 g, 6.1 mmol) was added and stirred for 10 min. The solvent was removed under reduced pressure, and the resulting white solid was washed with acetone (10 mL) and dichloromethane (10 mL) to give the purified product as a white solid in an 85% yield (1.0 g). ^1^H NMR (300 MHz, D_2_O) δ 4.57 (dd, *J* = 8.9, 4.0 Hz, 2H), 3.48 (dd, *J* = 14.2, 4.0 Hz, 2H), 3.23 (dd, *J* = 14.2, 8.9 Hz, 2H), 2.11 (s, 6H); ^13^C NMR (75 MHz, D_2_O) δ 176.3, 173.7, 53.9, 40.2, 22.0. ESI-MS: calcd. for C_10_H_15_N_2_O_6_S_3_^–^, 355.0092; found, 355.0098.

### 4.6 *N*-Acetylcysteine Tetrasulfide

A literature procedure was followed with slight modification.^41^*N*-Acetylcysteine (1.6 g, 9.8 mmol) was added to an oven-dried flask and dissolved in dry THF (25 mL) and placed in an ice bath. S_2_Cl_2_ (0.63 g, 4.6 mmol) was added to dry THF (5 mL) and, using an addition funnel, slowly added to *N*-acetylcysteine over 15 min. The reaction was removed from the ice bath and stirred for an additional 14 h. The solvent was removed under reduced pressure to give the pure product as a white solid in an 81% yield (1.4 g). ^1^H NMR (300 MHz, D_2_O) δ 4.81 (2H), 3.60 (dd, *J* = 14.4, 4.4 Hz, 2H), 3.35 (dd, *J* = 14.4, 9.0 Hz, 2H), 2.06 (s, 6H); ^13^C NMR (75 MHz, D_2_O) δ 174.2, 173.5, 52.0, 39.2, 21.7.

#### 4.6.1 Stability Studies of Trisulfides and Polysulfides

*N*-Acetylcysteine trisulfide (26.5 mg, 66 μmol) was dissolved in 600 μL of D_2_O. Butylamine (5.0 mg, 68 μmol) was added, and the ^1^H NMR spectra were taken periodically to track degradation.

*N*-Acetylcysteine trisulfide (26.5 mg, 66 μmol) was dissolved in 600 μL of D_2_O. Triethylamine (8.0 mg, 79 μmol) was added, and the ^1^H NMR spectra were taken periodically to track degradation.

*N*-Acetylcysteine trisulfide (26.5 mg, 66 μmol) was dissolved in 600 μL of D_2_O. Acetonitrile (3.0 mg, 73 μmol) was added, and the ^1^H NMR spectra were taken periodically to track degradation.

*N*-Acetylcysteine trisulfide (31.4 mg, 78 μmol) was dissolved in 600 μL of D_2_O. Benzamide (9.4 mg, 78 μmol) was added, and the ^1^H NMR spectra were taken periodically to track degradation.

*N*-Acetylcysteine trisulfide (15.0 mg, 38 μmol) was dissolved in 600 μL of D_2_O. ZnSO_4_ (11.8 mg, 73 μmol) was added, and the ^1^H NMR spectra were taken periodically to track degradation.

*N*-Acetylcysteine trisulfide (15.0 mg, 38 μmol) was dissolved in 600 μL of D_2_O. FeSO_4_·7H_2_O (11.6 mg, 41 μmol) was added, and the ^1^H NMR spectra were taken periodically to track degradation.

*N*-Acetylcysteine trisulfide (24.3 mg, 61 μmol) was dissolved in 500 μL of D_2_O and placed in a 40 °C oil bath. The ^1^H NMR spectra were taken periodically to track degradation.

*N*-Acetylcysteine trisulfide (24.3 mg, 61 μmol) was dissolved in 500 μL of D_2_O and placed in a 60 °C oil bath. The ^1^H NMR spectra were taken periodically to track degradation.

*N*-Acetylcysteine trisulfide (32.1 mg, 80 μmol) was dissolved in 600 μL of CD_3_OD. The ^1^H NMR spectra were taken periodically to track degradation.

*N*-Acetylcysteine trisulfide (14.4 mg, 36 μmol) was dissolved in 3.6 mL of D_2_O buffered to pH 7.4. Butylamine (11 μL, 110 μmol) was added and the contents were heated to 37 °C. The ^1^H NMR spectra were taken periodically to track degradation.

Cysteine trisulfide (5.5 mg, 20 μmol) was added to 600 μL of D_2_O. The solution was filtered to remove undissolved particles. Butylamine (1.8 mg 25 μmol) was added, and integrations in the ^1^H NMR spectrum showed that the ratio of cysteine trisulfide to butylamine was 1:2. The ^1^H NMR spectra were taken periodically to track degradation.

Cysteine trisulfide (4.3 mg, 16 μmol) was added to 600 μL of D_2_O. The solution was filtered to remove undissolved particles. Triethylamine (2.5 mg 25 μmol) was added, and integrations in the ^1^H NMR spectrum showed that the ratio of cysteine trisulfide to triethylamine was 1:2. The ^1^H NMR spectra were taken periodically to track degradation.

Cysteine trisulfide (5.1 mg, 19 μmol) was added to 600 μL of D_2_O. Solution was filtered to remove undissolved particles. Acetonitrile (1.1 mg 26 μmol) was added, and the integrations in the ^1^H NMR spectrum showed a 3 equiv excess of acetonitrile. The ^1^H NMR spectra were taken periodically to track degradation.

Cysteine trisulfide (6.8 mg, 25 μmol) was added to 600 μL of D_2_O. Solution was filtered to remove undissolved particles. Benzamide (3.1 mg 25 μmol) was added, and the integrations in the ^1^H NMR spectrum showed a 3 equiv excess of benzamide. The ^1^H NMR spectra were taken periodically to track degradation.

*N*-Acetylcysteine tetrasulfide (17.2 mg, 44 μmol) was dissolved in 600 μL of D_2_O. Butylamine (9.9 mg, 135 μmol) was added, and the ^1^H NMR spectra were taken periodically to track degradation.

*N*-Acetylcysteine tetrasulfide (17.2 mg, 44 μmol) was dissolved in 600 μL of D_2_O. Triethylamine (13.5 mg, 135 μmol) was added, and the ^1^H NMR spectra were taken periodically to track degradation.

*N*-Acetylcysteine tetrasulfide (17.2 mg, 44 μmol) was dissolved in 600 μL of D_2_O. Acetonitrile (1.9 mg, 47 μmol) was added, and the ^1^H NMR spectra were taken periodically to track degradation.

*N*-Acetylcysteine tetrasulfide (17.2 mg, 44 μmol) was dissolved in 600 μL of D_2_O. Benzamide (5.5 mg, 45 μmol) was added, and the ^1^H NMR spectra were taken periodically to track degradation.

*N*-Acetylcysteine tetrasulfide (8.0 mg, 21 μmol) was dissolved in 600 μL of D_2_O. The ^1^H NMR spectra were taken periodically to track degradation.

*N*-Acetylcysteine tetrasulfide (10.0 mg, 26 μmol) was dissolved in 600 μL of D_2_O and placed in a 40 °C oil bath. The ^1^H NMR spectra were taken periodically to track degradation.

#### 4.6.2 Determination of the Insoluble Solid as Elemental Sulfur

This general description was followed to determine that the solid precipitate was elemental sulfur. *N*-Acetylcysteine trisulfide was dissolved in water. An excess of triethylamine was added and stirred for 1 day. The insoluble solid was filtered and washed with water. The off-white solid (20 mg) was dried under reduced pressure. Both ^1^H and ^13^C NMR spectra did not show any peaks. The solid was dissolved in 5 mL of toluene, triphenylphosphine (164 mg, 0.63 mmol) was added, and the solution was stirred overnight. Toluene was removed under reduced pressure to yield an off-white solid. The ^31^P NMR spectrum (CDCl_3_, 300 MHz) showed the starting material (δ 5.41) and triphenylphosphine sulfide (δ 43.4).

#### 4.6.3 Investigation of the Degradation Mechanism of *N*-Acetylcysteine Trisulfide in the Presence of an Amine

*N*-Acetylcysteine trisulfide (51.7 mg, 0.13 mmol) was dissolved in 2 mL of methanol. *N*-Ethylmaleimide (27.5 mg, 0.22 mmol) and butylamine (13 μL, 0.13 mmol) were added. Mass spectroscopy was used to identify degradation products.

*N*-Acetylcysteine trisulfide (20.5 mg, 51 μmol) and cysteine trisulfide (13.6 mg, 51 μmol) were dissolved in 2 mL of DI H_2_O. Butylamine (5 μL, 51 μmol) was added. High-resolution mass spectroscopy was used to identify degradation products.

#### 4.6.4 Investigation of the Equilibrium between Disulfides and Polysulfides in the Presence of Sulfur and an Amine

*N*-Acetylcysteine disulfide (9.3 mg, 23 μmol) was dissolved in 600 μL of D_2_O. Elemental sulfur (6.8 mg, 0.21 mmol) and butylamine (18.5 mg, 0.25 mmol) were added, and the ^1^H NMR spectra were periodically obtained to track the equilibrium.

#### 4.6.5 Kinetic Study of the Degradation of Trisulfides in the Presence of a Thiol

Cysteine trisulfide (0.7 mg, 2.6 μmol) was dissolved in 500 μL of D_2_O buffered to pH 7.0, yielding a 5 mM solution. Cysteine (1.5 mg, 12.4 μmol) was added, and the ^1^H NMR spectra were periodically obtained to track degradation.

Glutathione trisulfide (1.6 mg, 2.6 μmol) was added to 500 μL of D_2_O buffered to a pH of 7.0, yielding a 5 mM solution. Glutathione (3.8 mg, 12.4 μmol) was added, and the ^1^H NMR spectra were obtained periodically to track degradation.

*N*-Acetylcysteine trisulfide (1.0 mg, 2.6 μmol) was dissolved in 500 μL of D_2_O buffered to pH 7.0, yielding a 5 mM solution. *N*-Acetylcysteine (2.1 mg, 12.4 μmol) was added, and the ^1^H NMR spectra were obtained periodically to track degradation.

Cysteine trisulfide (0.7 mg, 2.6 μmol) was dissolved in 500 μL of D_2_O buffered to pH 7.0, yielding a 5 mM solution. *tert*-Butanol (0.8 μL, 2.6 μmol) was added as an internal standard. 1-Propanethiol (1.13 μL, 12.4 μmol) was added, and the ^1^H NMR spectra were periodically obtained to track degradation.

Glutathione trisulfide (1.6 mg, 2.6 μmol) was dissolved in 500 μL of D_2_O buffered to pH 7.0, yielding a 5 mM solution. *tert*-Butanol (0.8 μL, 2.6 μmol) was added as an internal standard. 1-Propanethiol (1.13 μL, 12.4 μmol) was added, and the ^1^H NMR spectra were periodically obtained to track degradation.

*N*-Acetylcysteine trisulfide (1.1 mg, 2.6 μmol) was dissolved in 500 μL of D_2_O buffered to pH 7.0, yielding a 5 mM solution. *tert*-Butanol (0.8 μL, 2.6 μmol) was added as an internal standard. 1-Propanethiol (1.13 μL, 12.4 μmol) was added, and the ^1^H NMR spectra were periodically obtained to track degradation.

### 4.7 H_2_S Measurement Using an H_2_S Electrode

H_2_O buffered with phosphate (1 M) at pH 6.7 was added to a glass jar. H_2_S was measured to calibrate the instrument and establish a baseline. The corresponding tri- and tetrasulfides were added. After 30 min, cysteine was added and the pH and concentration of H_2_S were measured every 2 s and logged into a spreadsheet.

## Supporting Information Available

NMR spectra of selected chemicals and reactions; kinetics of reactions (PDF)
