# Supplementary material for: Stabilities of Three Key Biological Trisulfides with Implications for Their Roles in the Release of Hydrogen Sulfide and Bioaccumulation of Sulfane Sulfur
Source: ACS Omega. 2022 Mar 22;7(13):11440–51. doi: 10.1021/acsomega.2c00736 (PMC8992272; doi:10.1021/acsomega.2c00736)
Supplement: Supplementary file 1 — ao2c00736_si_001.pdf [file ao2c00736_si_001.pdf]

Supporting Information

Stabilities of Three Key Biological Trisulfides with Implications for Their Roles in the Release of Hydrogen Sulfide and Bioaccumulation of Sulfane Sulfur

Eric M. Brown and Ned B. Bowden*
Department of Chemistry
University of Iowa, Iowa City IA, 52242

Ned-bowden@uiowa.edu

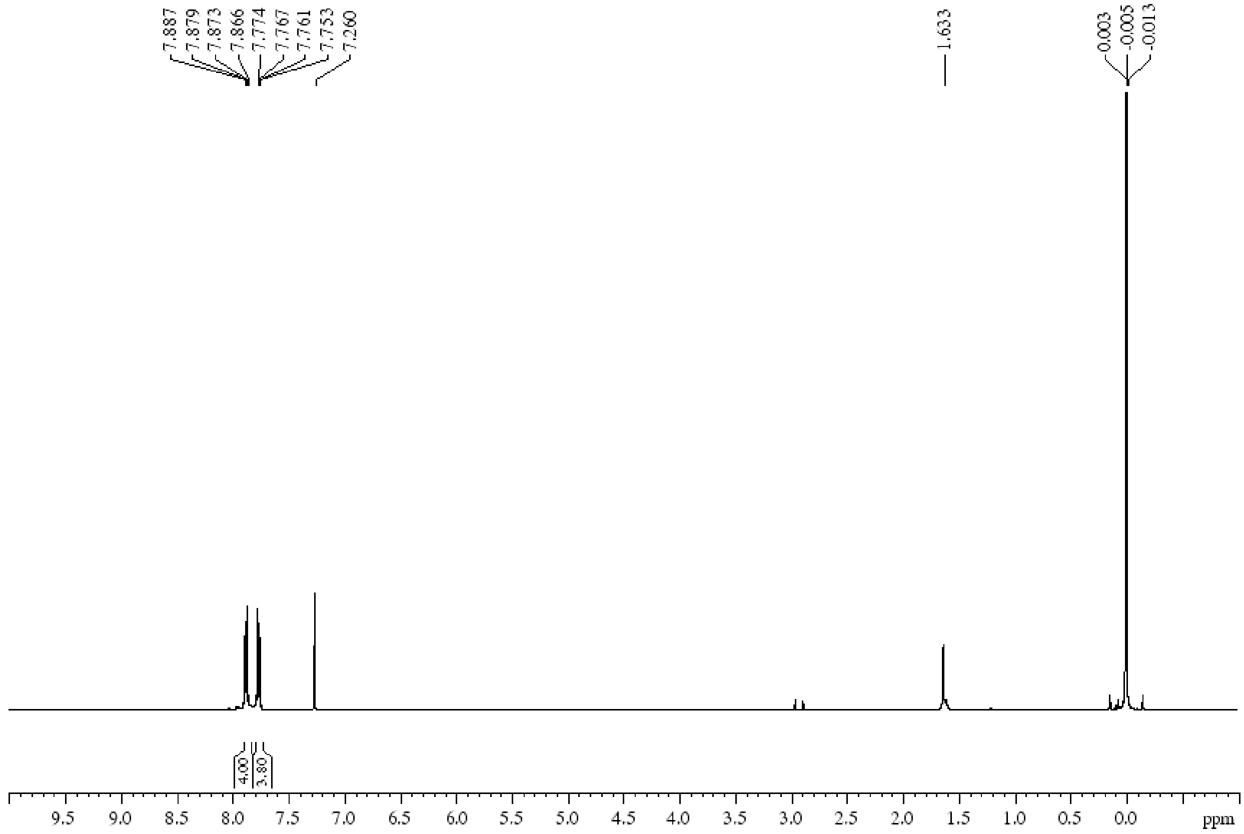

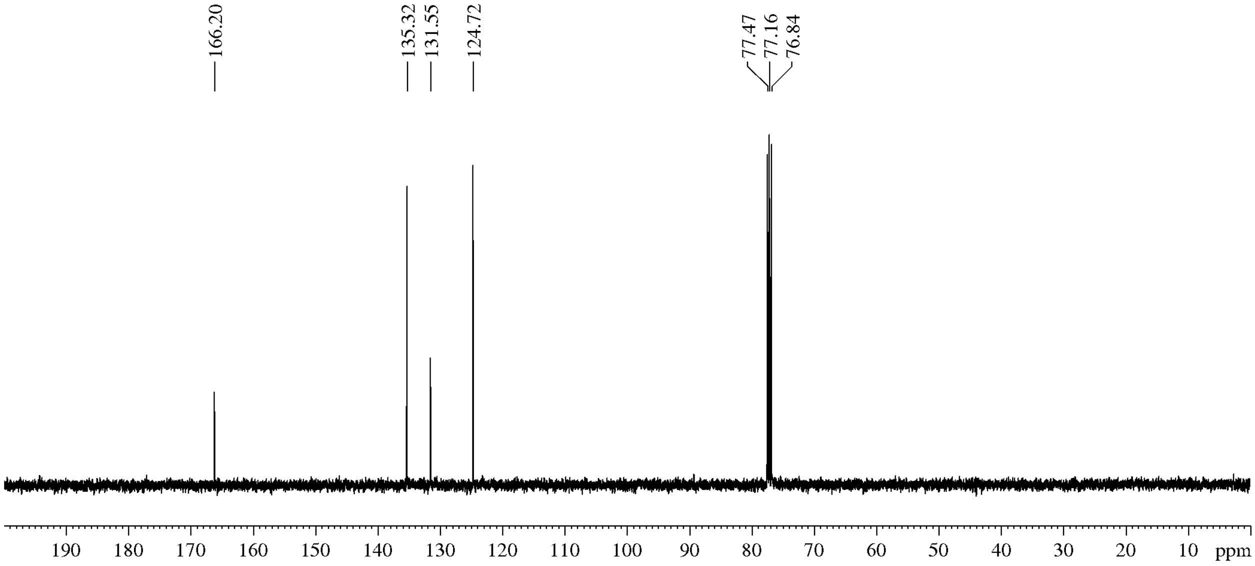

Figure S1. ^{1}H and ^{13}C NMR spectra of 2,2'-thiobis(isoindoline-1,3-dione) (monosulfide transfer reagent **1**) in $CDCl_3$.

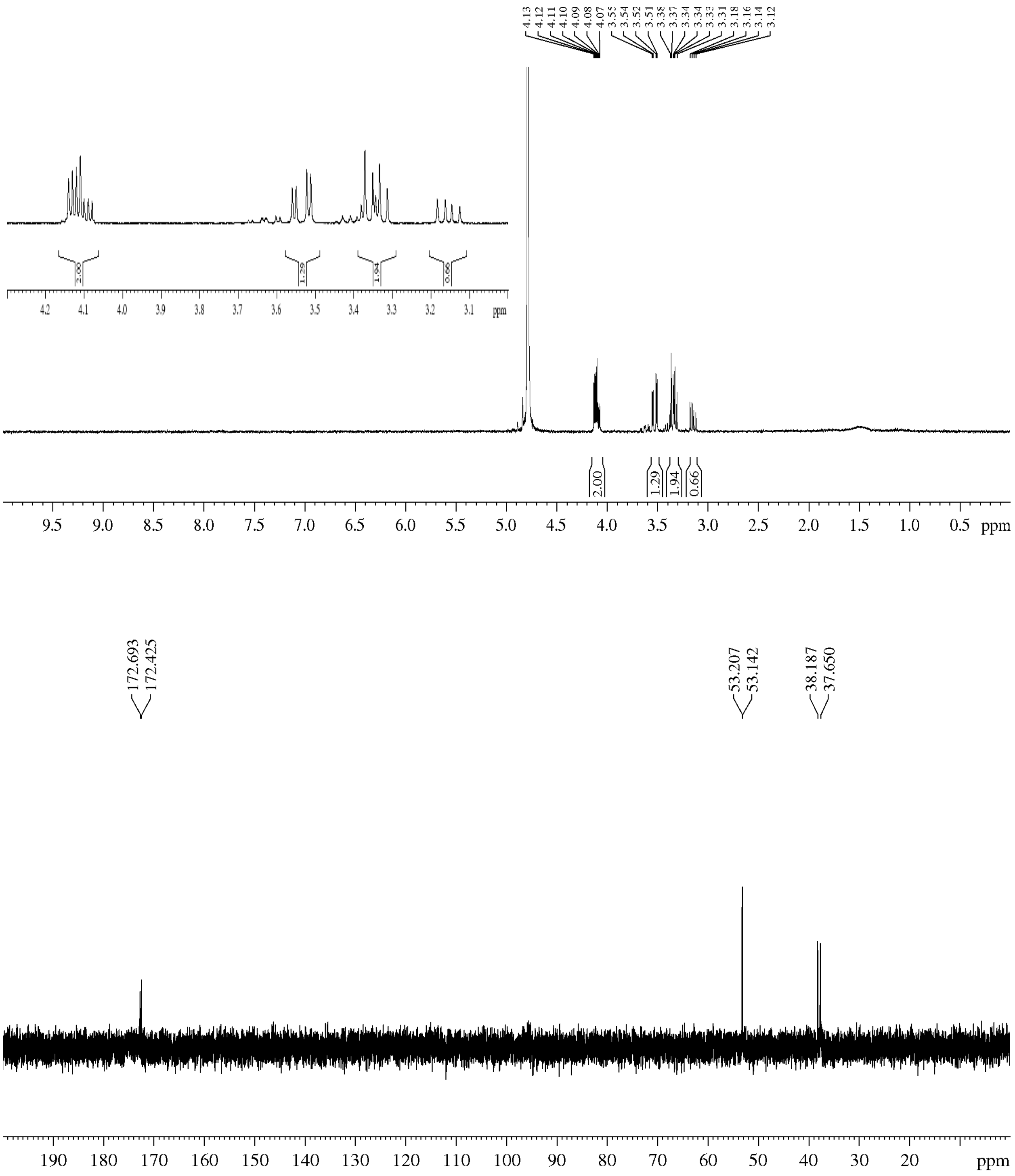

Figure S2. ^{1}H and ^{13}C NMR spectra of cysteine trisulfide in D_2O.

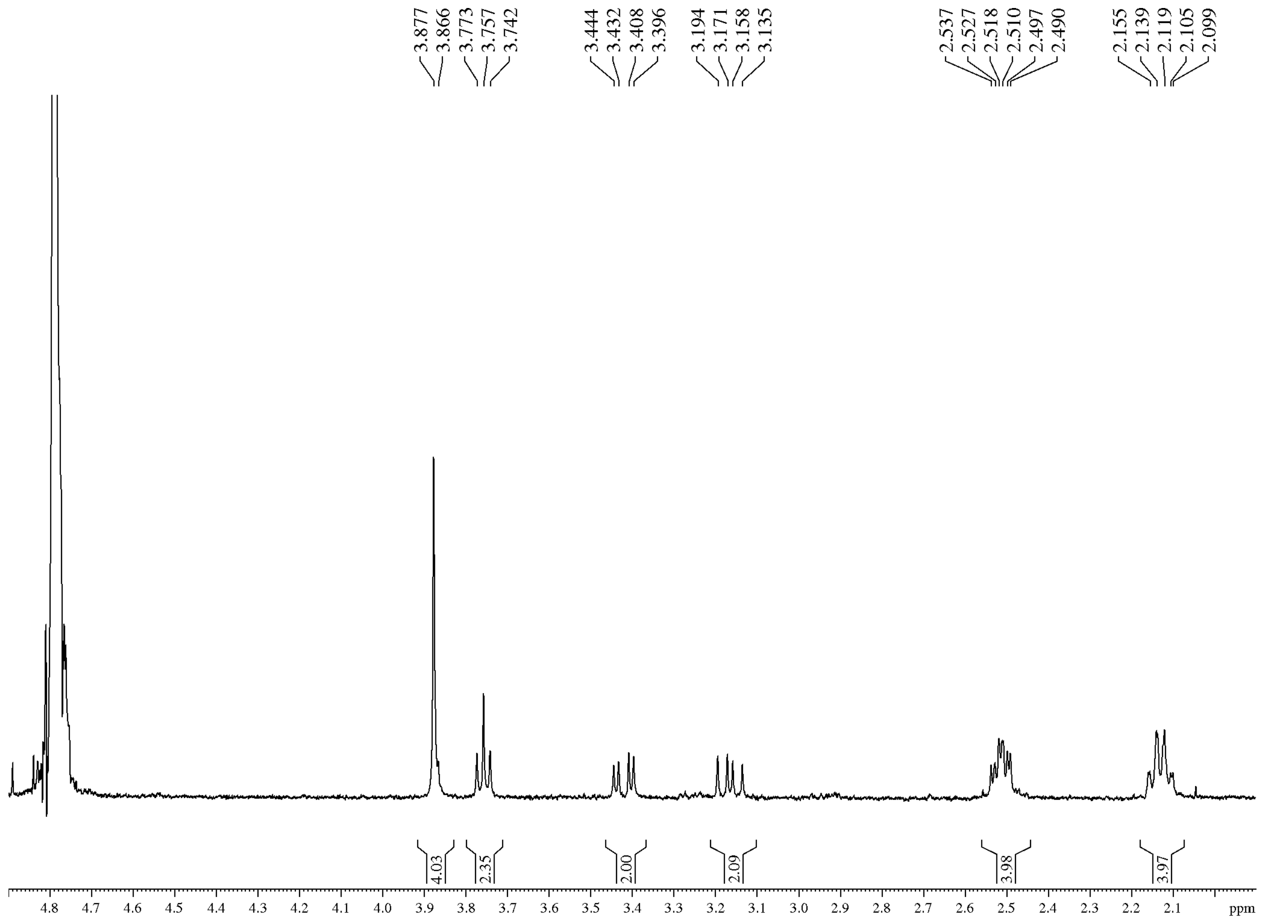

Figure S3. ^{1}H NMR spectrum of glutathione trisulfide in D_2O.

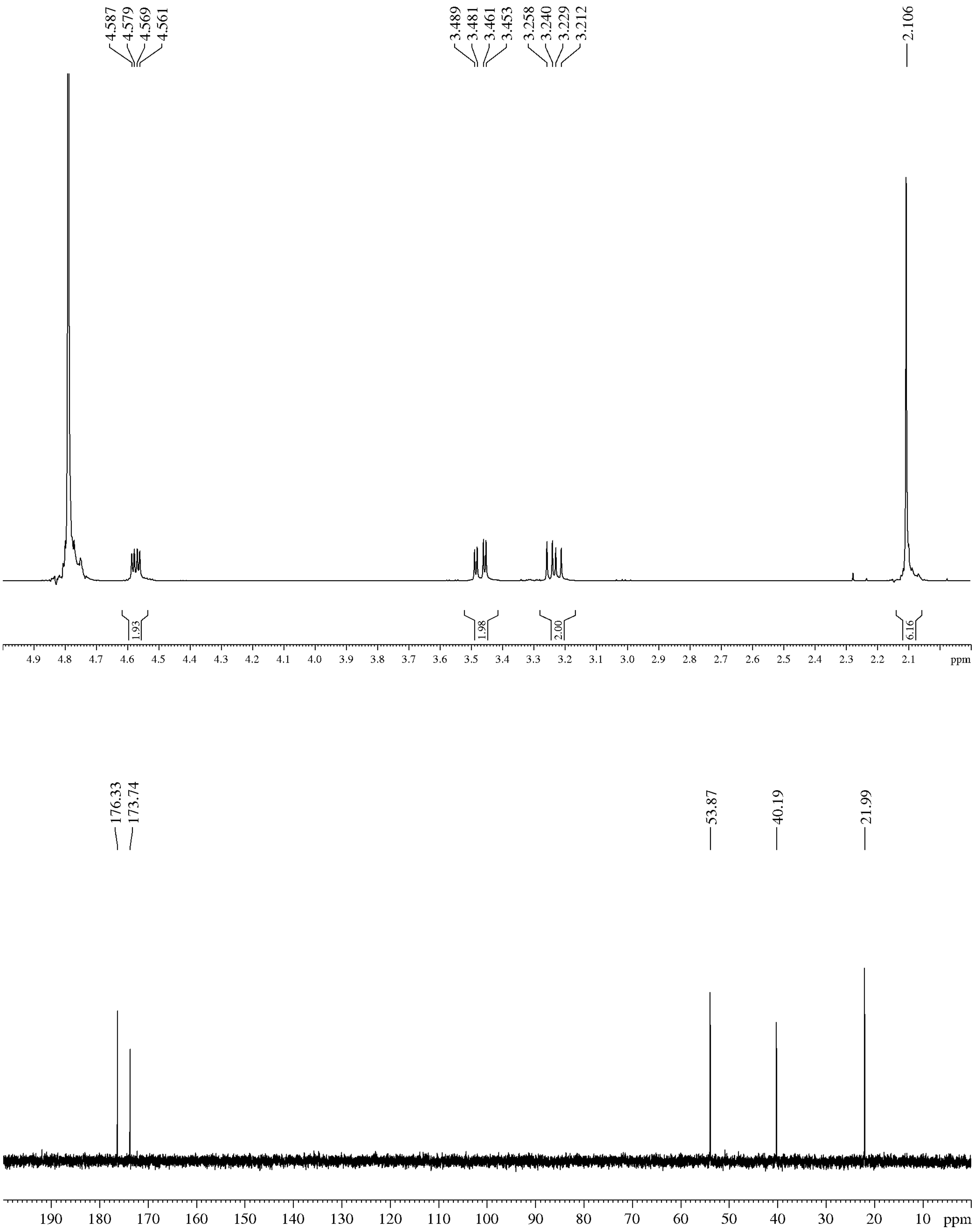

Figure S4. ^{1}H and ^{13}C NMR spectra of NAC trisulfide in D_2O.

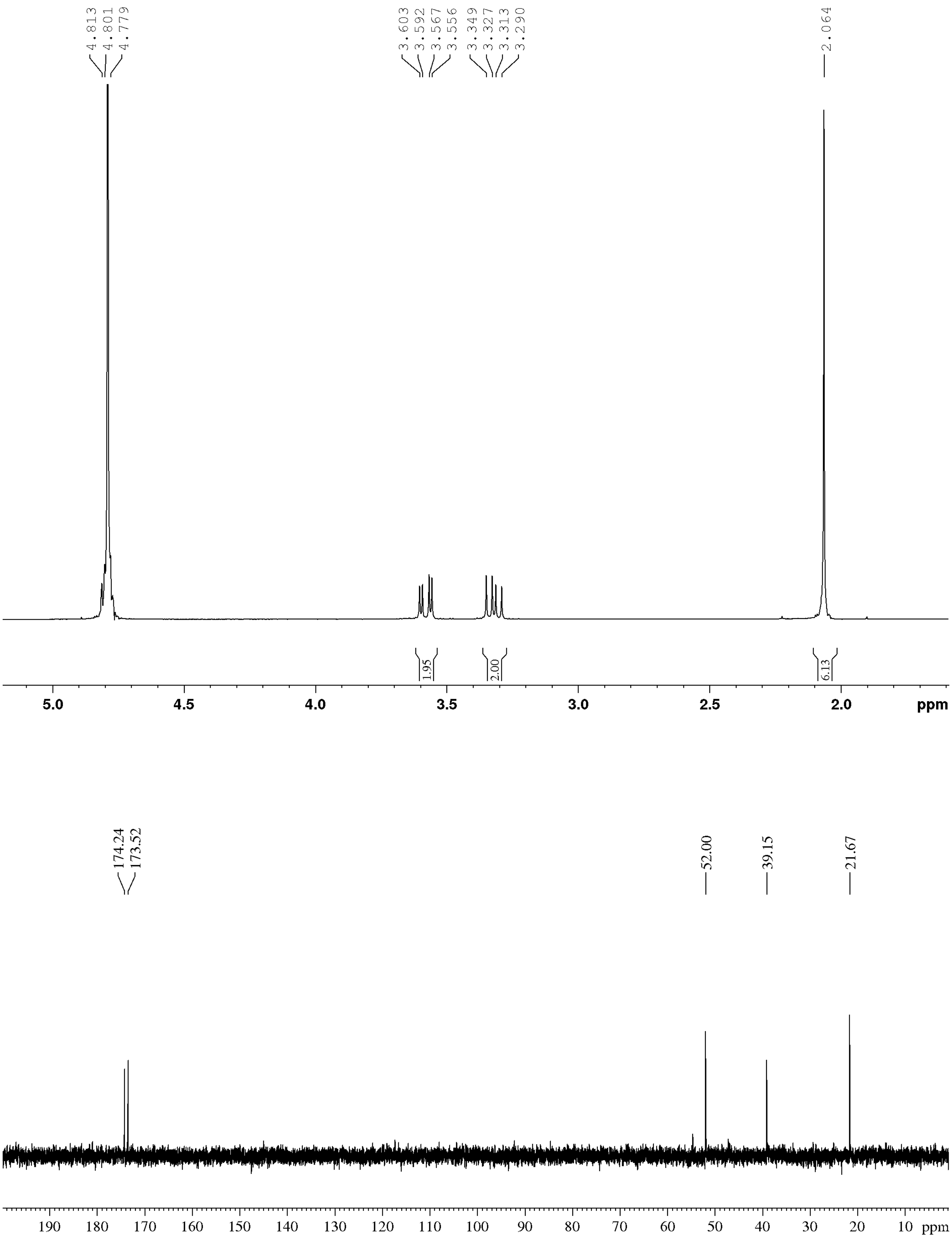

Figure S5. ^{1}H and ^{13}C NMR spectra of NAC tetrasulfide in D_2O.

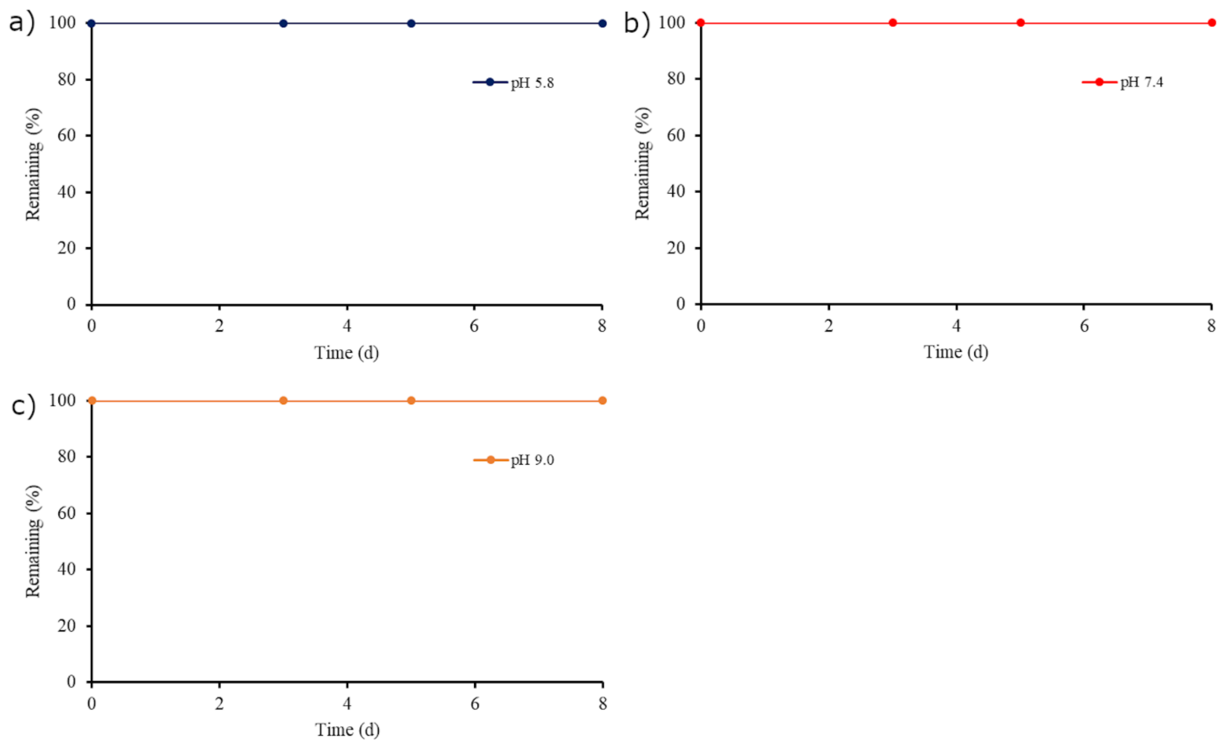

Figure S6. NAC trisulfide showed no degradation after 8 days as observed by ^{1}H NMR spectroscopy in water at pH values of a) 5.8, b) 7.4, and c) 9.0.

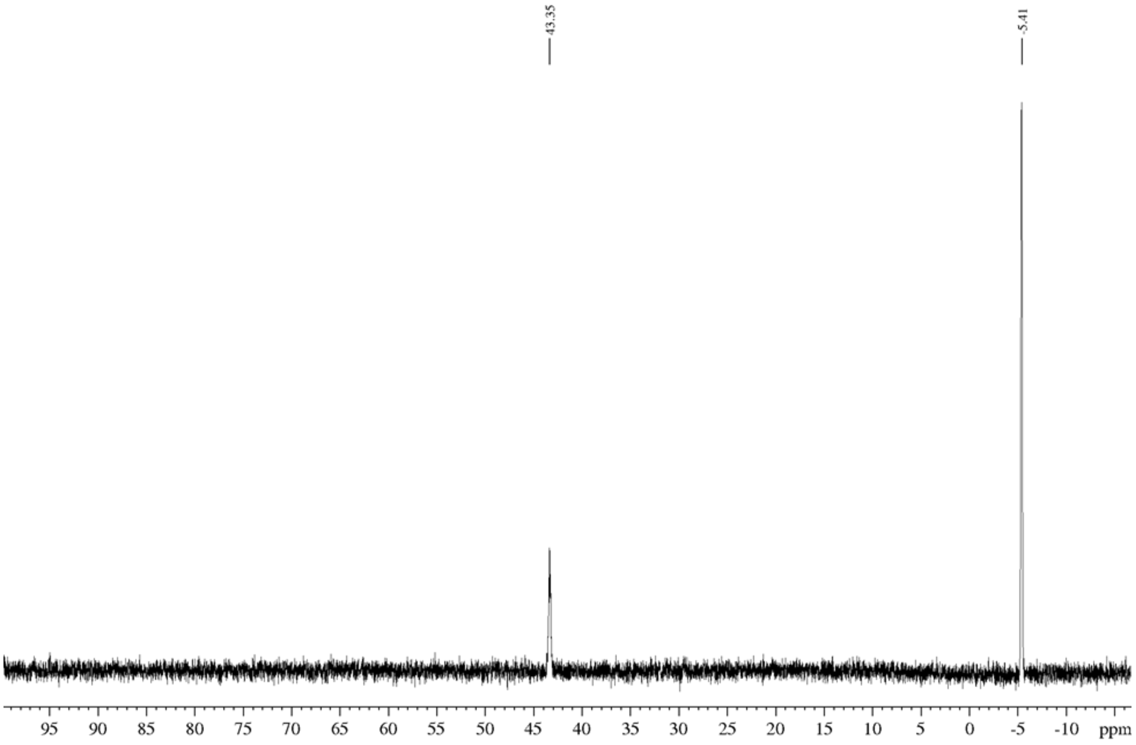

Figure S7. ^{31}P NMR spectrum of the reaction between triphenyl phosphine (5.41 ppm) and sulfur generated triphenyl phosphine sulfide (43.35 ppm).

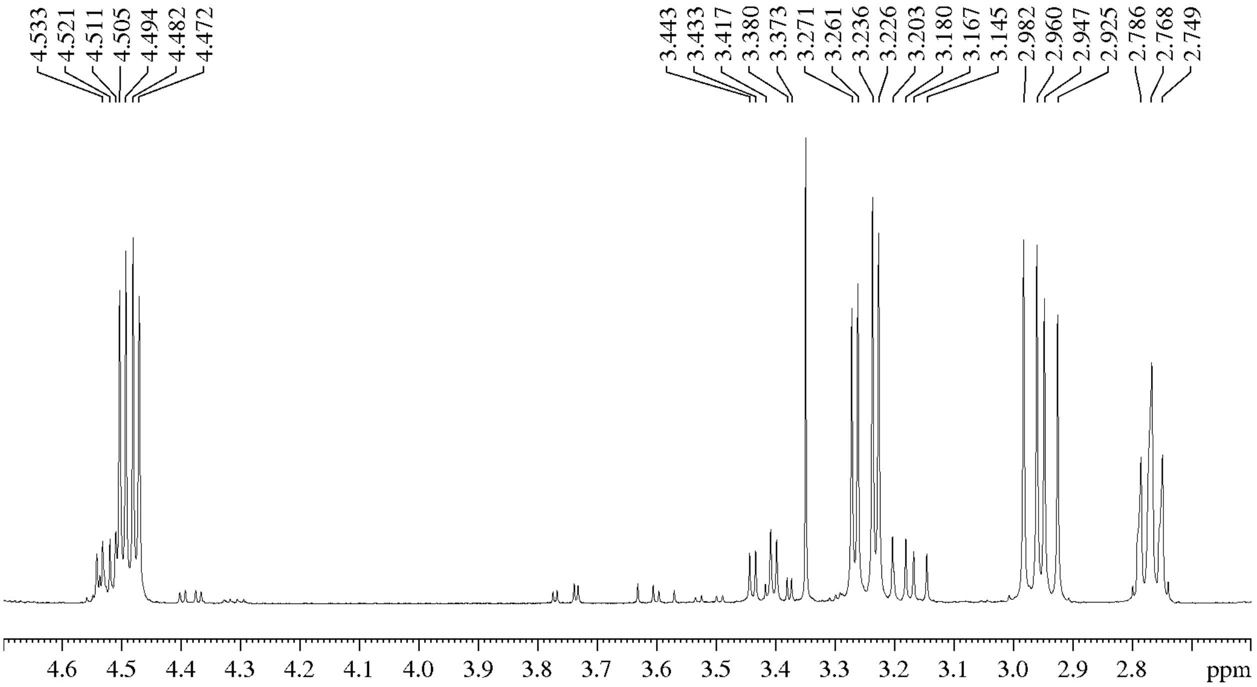

Figure S8. ^{1}H NMR spectrum after 4 days shows NAC disulfide converted to tri, tetra, and polysulfides in the presence of butyl amine and elemental sulfur in D_2O.

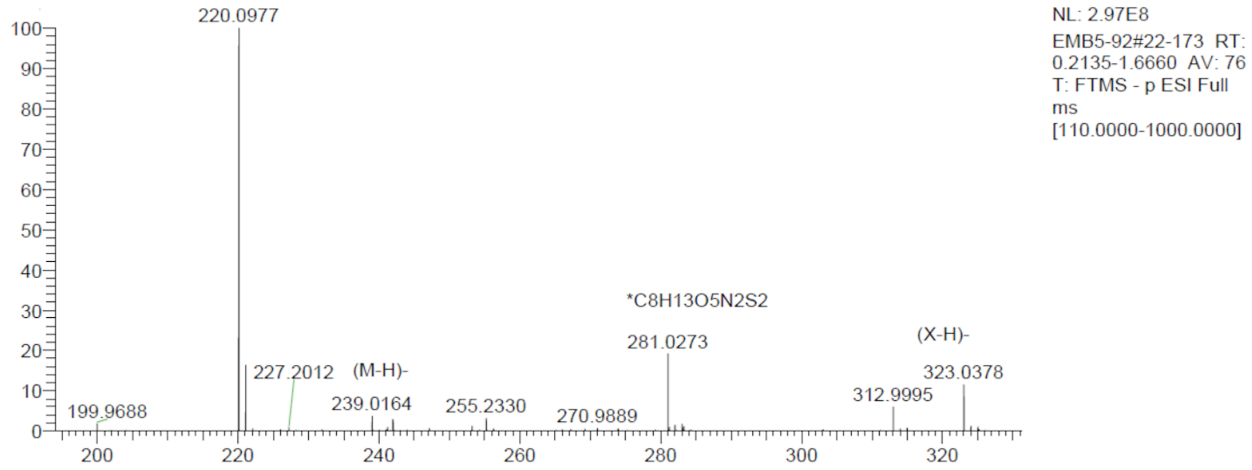

Figure S9. High resolution mass spectroscopy spectrum of the reaction between NAC trisulfide, cysteine trisulfide, and butylamine shows the formation of the mixed disulfide of NAC and cysteine. Calculated: 281.0266. Found: 281.0273.

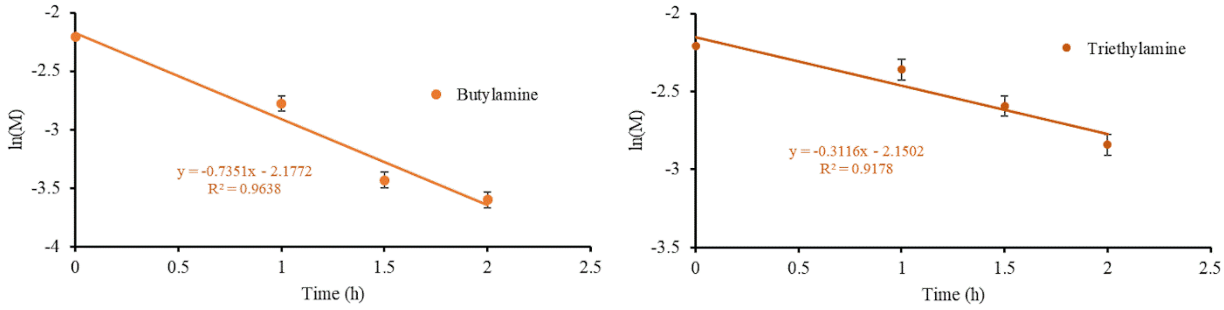

Figure S10. Graphs of the degradation of NAC trisulfide in the presence of butylamine (left) and triethylamine (right) fit to pseudo first order reaction kinetics. Due to the equilibrium, only early data points were used to provide an estimated rate constant. The estimated rate constants for the degradation of NAC trisulfide in the presence of butylamine and triethylamine were 0.74 h^{-1} and 0.31 h^{-1}, respectively. Error bars are ± instrument error.

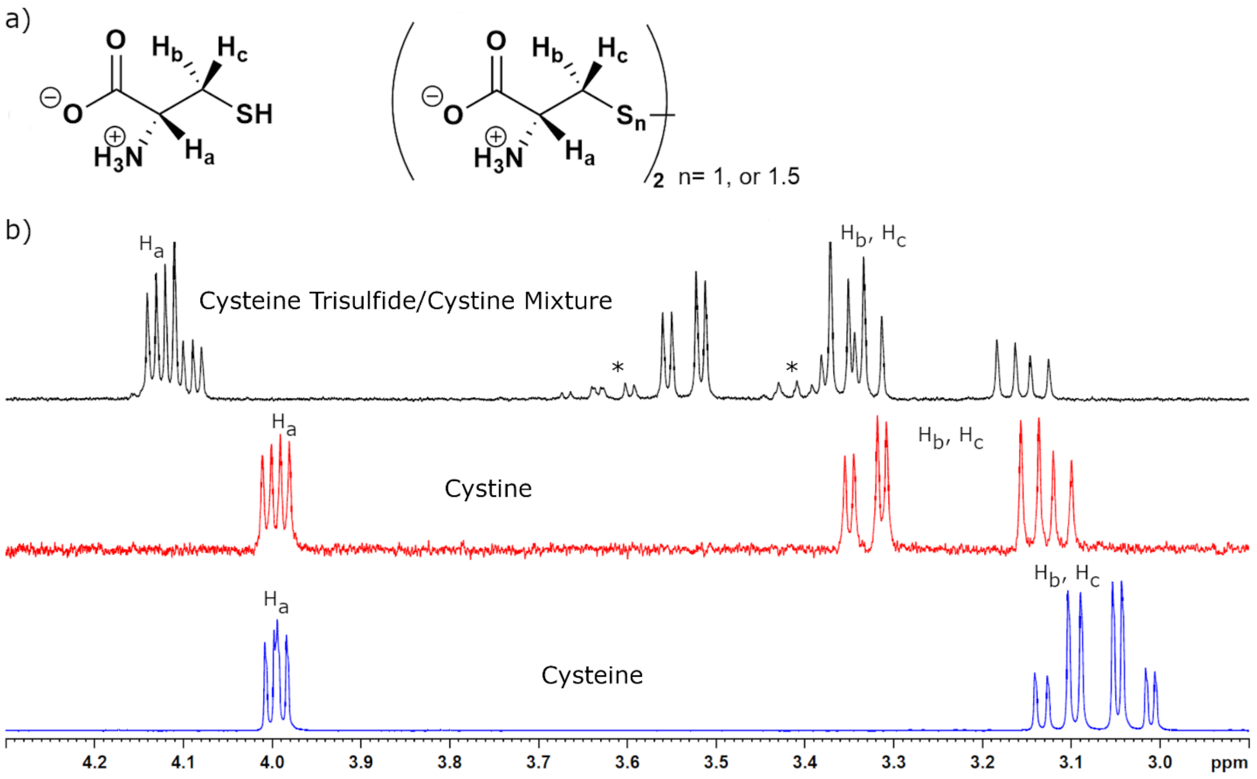

Figure S11. a) The general structure of cysteine and cysteine polysulfides are shown. b) Relevant regions of ^{1}H NMR spectra of cysteine (bottom), cystine (middle), and cysteine di- tri- and tetrasulfide mixture mixture (top) with the tetrasulfide labelled as “*”.

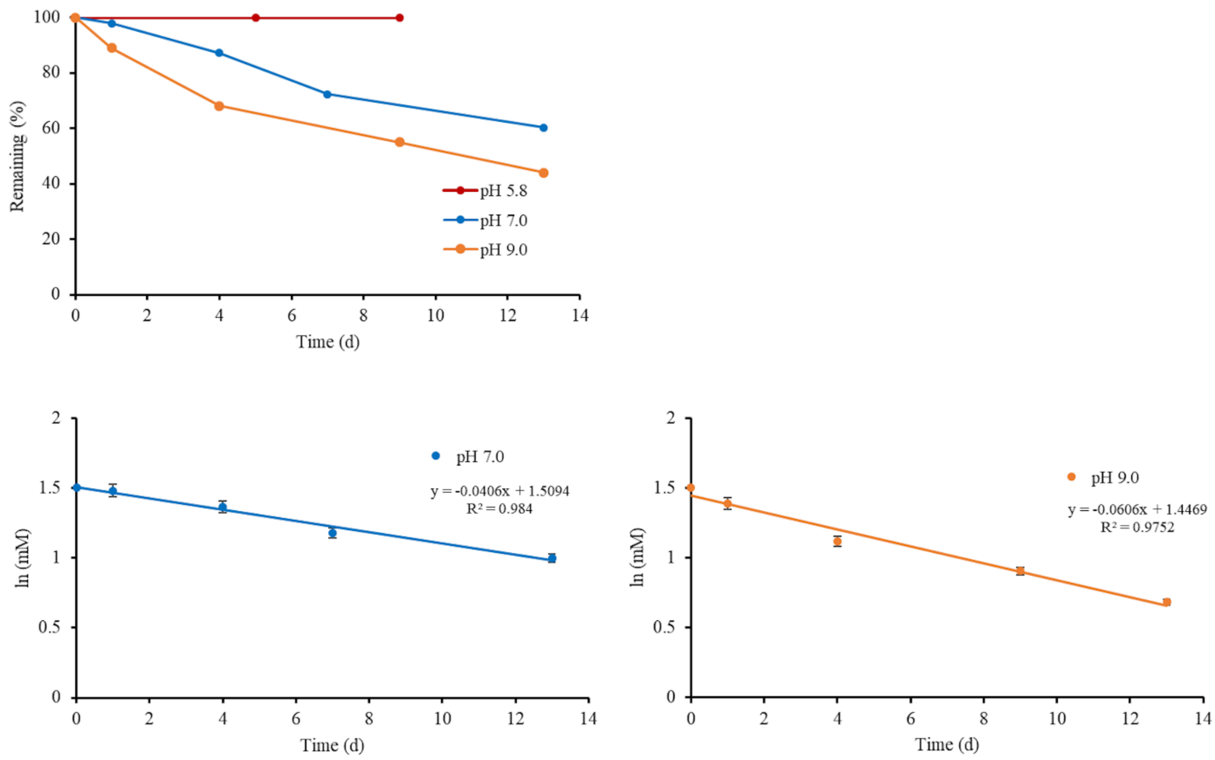

Figure S12. The stabilities of cysteine trisulfide at pH values of 5.8 (red), 7.0 (blue), and 9.0 (orange) were measured by ^{1}H NMR spectroscopy (top). Graphs of the degradation of cysteine trisulfide at pH 7.0 (bottom left) and pH 9.0 (bottom right). The rate constants for the degradation of cysteine trisulfide at pH 7.0 and 9.0 are 0.041 d^{-1} and 0.061 d^{-1}, respectively. Error bars are ± instrument error.

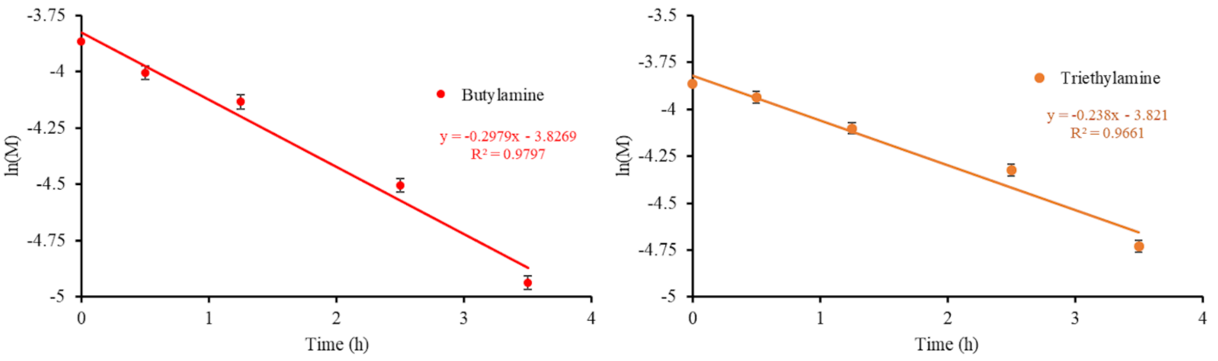

Figure S13. Graphs of the degradation of cysteine trisulfide in the presence of butylamine (left) and triethylamine (right) fit to pseudo first order reaction kinetics. Due to the equilibrium, only early data points were used to yield an estimated rate constant. The estimated rate constants for the degradation of cysteine trisulfide in the presence of butylamine and triethylamine are 0.30 h^{-1} and 0.24 h^{-1}, respectively. Error bars are ± instrument error.

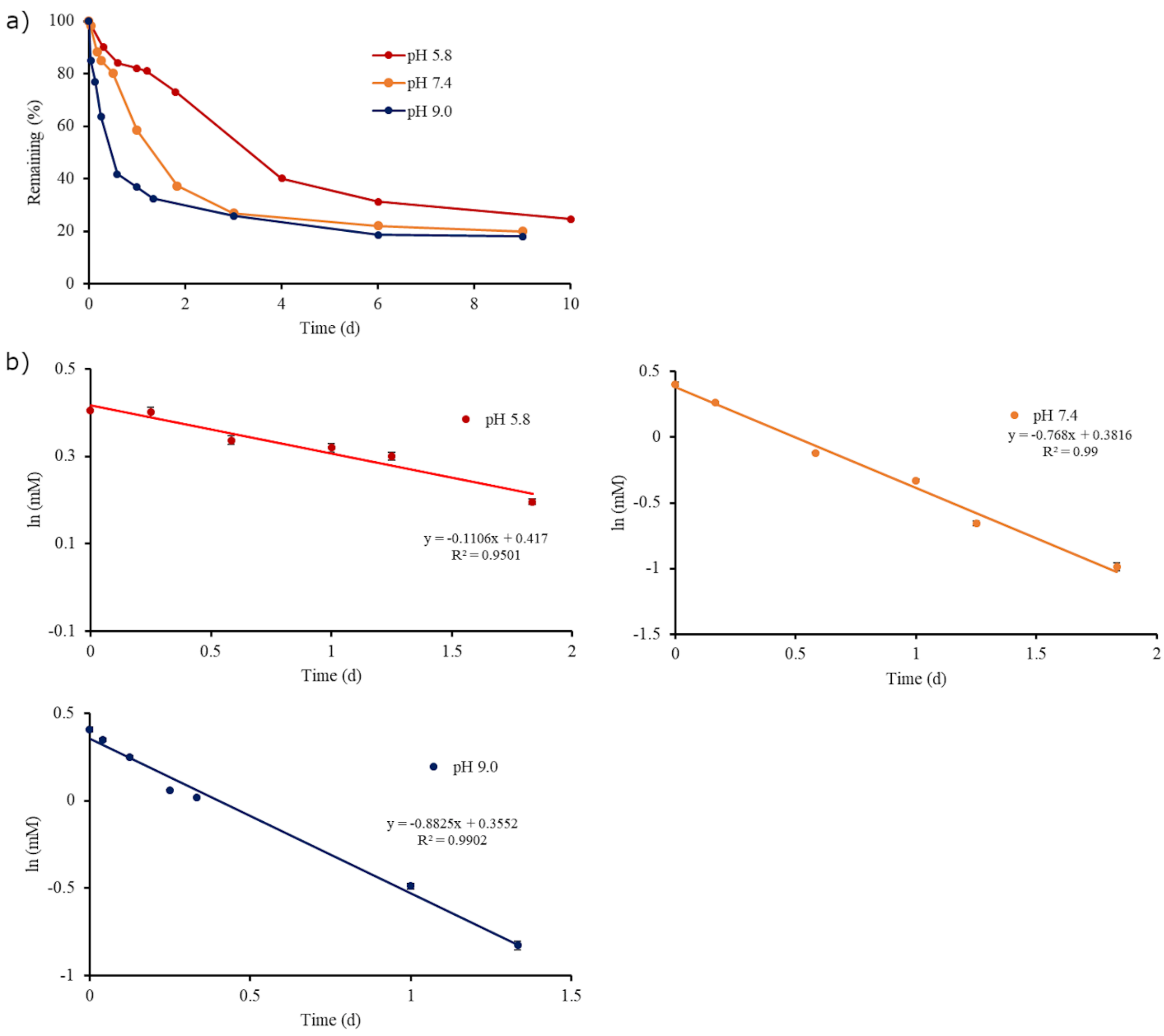

Figure S14. a) Stability of glutathione trisulfide at pH values of 5.8 (red), pH 7.4 (green), and pH 9.0 (blue) were measured by ^1H NMR spectroscopy. b) Graphs of the degradation of glutathione trisulfide at pH 5.8 (top left), pH 7.4 (top right), and pH 9.0 (bottom left). Due to the equilibrium, only early data points were used. The estimated rate constants for the degradation of glutathione trisulfide at pH 5.8, 7.0, and 9.0 are 0.11 d^{-1}, 0.77 d^{-1}, and 0.88 d^{-1}, respectively. Error bars are ± instrument error.

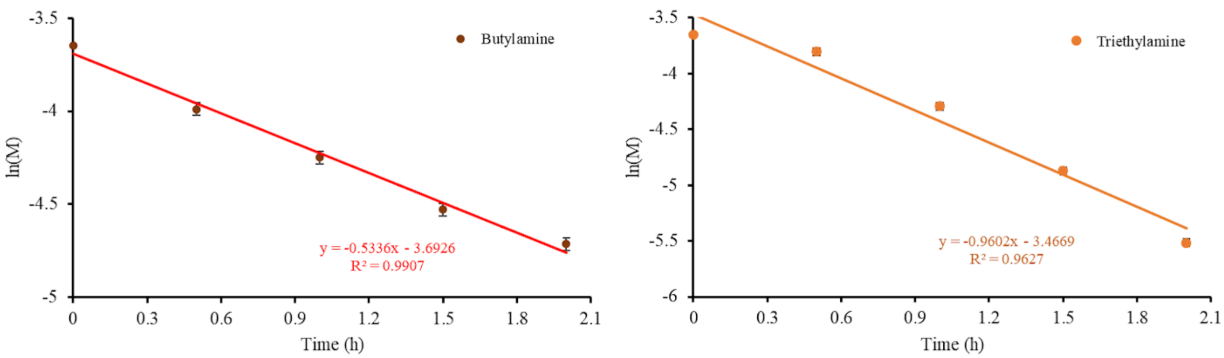

Figure S15. Graphs of the degradation of glutathione trisulfide in the presence of butylamine (left) and triethylamine (right) fit to pseudo first order reaction kinetics. Due to the equilibrium, only early data points were used. The estimated rate constants for the degradation of glutathione trisulfide in the presence of butylamine and triethylamine are 0.53 h^{-1} and 0.96 h^{-1}, respectively. Error bars are ± instrument error.

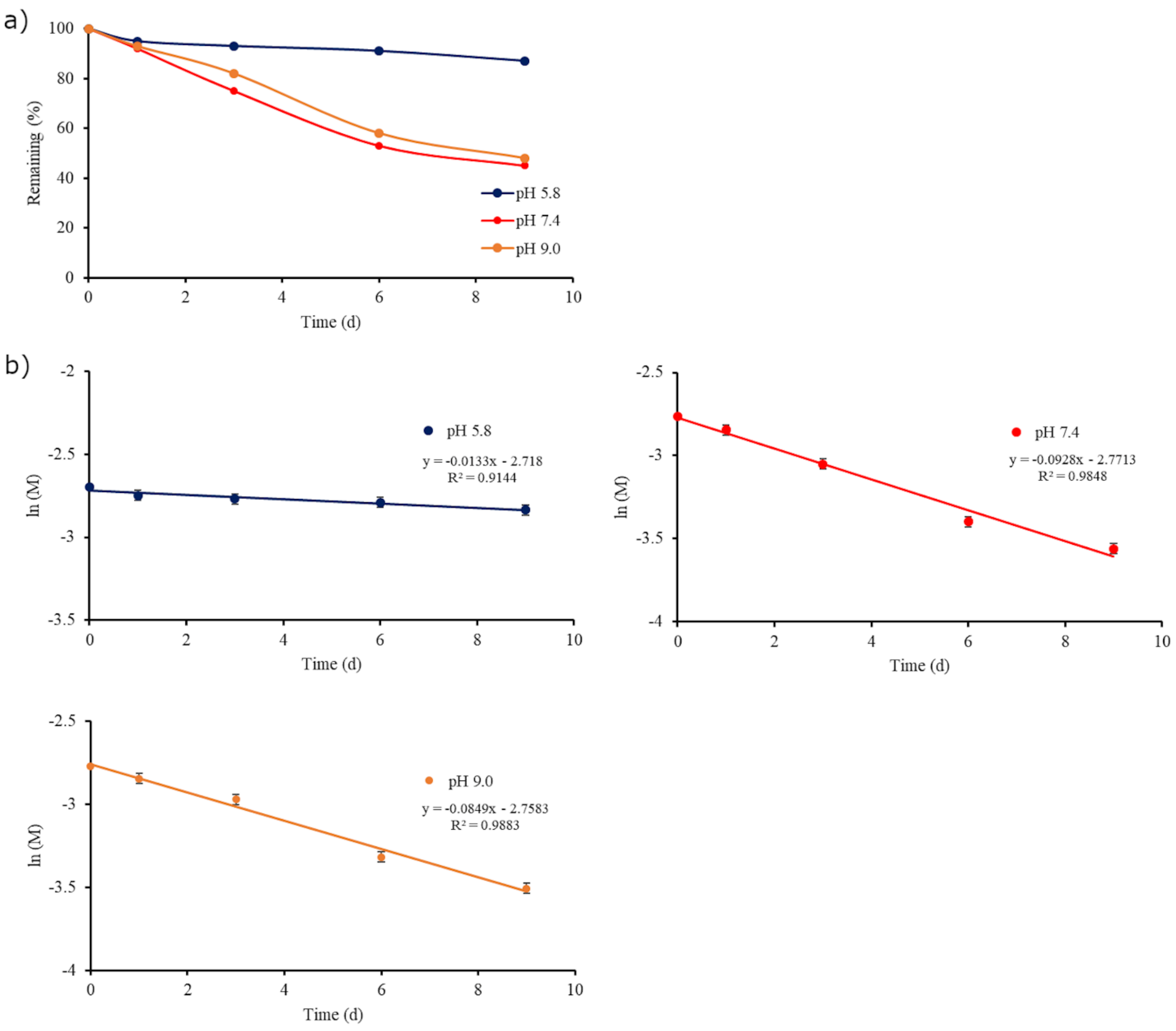

Figure S16. a) The stability of NAC tetrasulfide at pH values of 5.8 (blue), pH 7.4 (red), and pH 9.0 (orange) were measured by ^{1}H NMR spectroscopy. b) Graphs of the degradation of NAC tetrasulfide at pH 5.8 (top left), pH 7.4 (top right), and pH 9.0 (bottom left). The estimated rate constants for the degradation of NAC tetrasulfide at pH 5.8, 7.0, and 9.0 are 0.013 d^{-1}, 0.093 d^{-1}, and 0.085 d^{-1}, respectively. Error bars are ± instrument error.

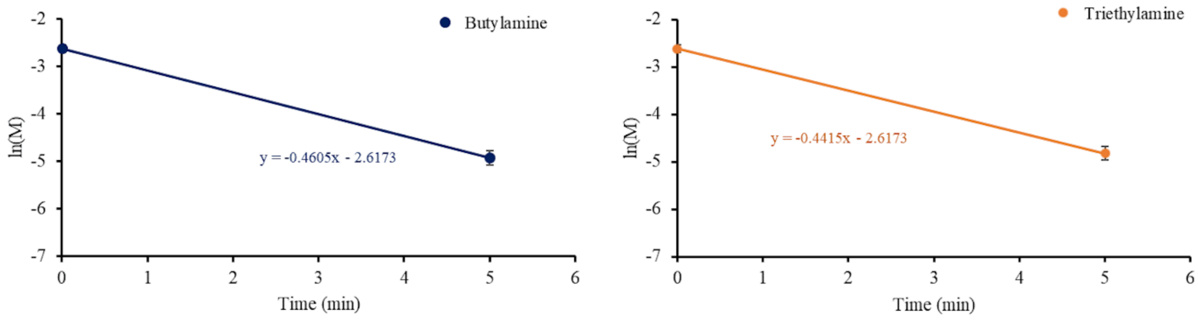

Figure S17. Graphs of the degradation of NAC tetrasulfide in the presence of butylamine (left) and triethylamine (right) fit to pseudo first order reaction kinetics. Due to the equilibrium, only early data points were used to yield an estimated rate constant. The estimated rate constants for the degradation of NAC tetrasulfide in the presence of butylamine and triethylamine are 0.46 min^{-1} and 0.44 min^{-1}, respectively. Error bars are ± instrument error.

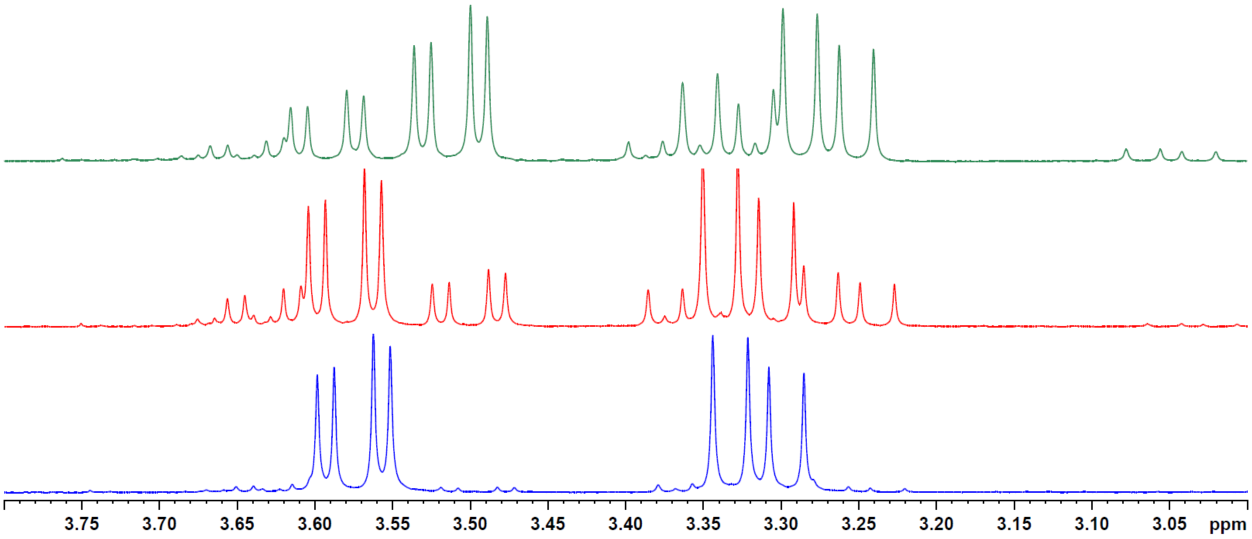

Figure S18. ^{1}H NMR spectra of NAC tetrasulfide (3.58 and 3.33 ppm) in D_2O after 1 day (bottom), 7 days (middle), and 35 days (top).

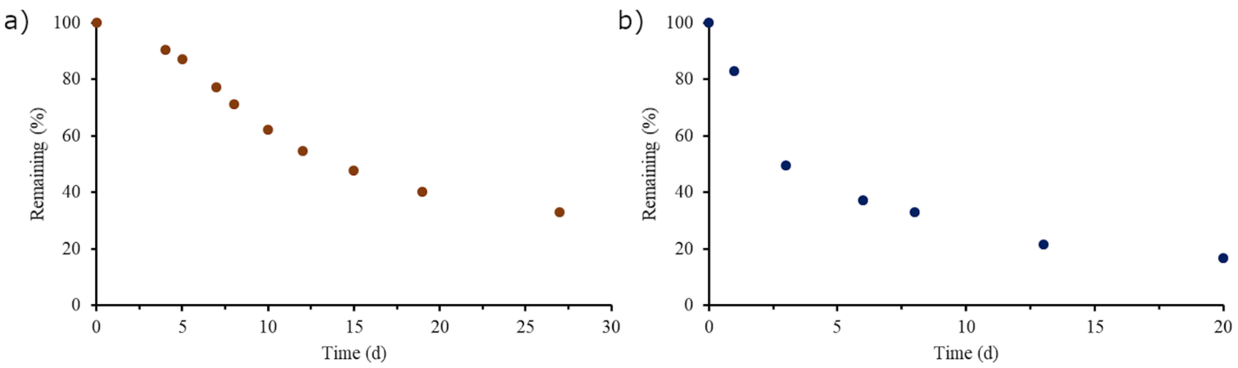

Figure S19. Graph of the disappearance of NAC tetrasulfide at a) room temperature and b) 40 °C.

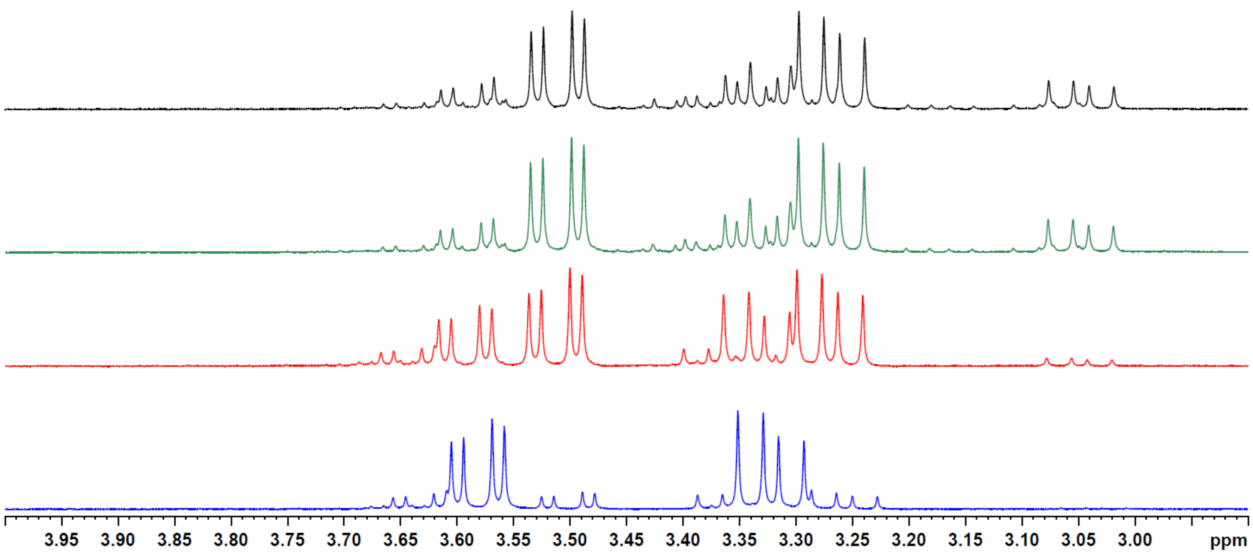

Figure S20. ^{1}H NMR spectra of NAC tetrasulfide (3.58 and 3.33 ppm) in D_2O at 40 °C after 1 day (bottom), 6 days, 35 days, and 63 days (top) showed the degradation slowed over time.

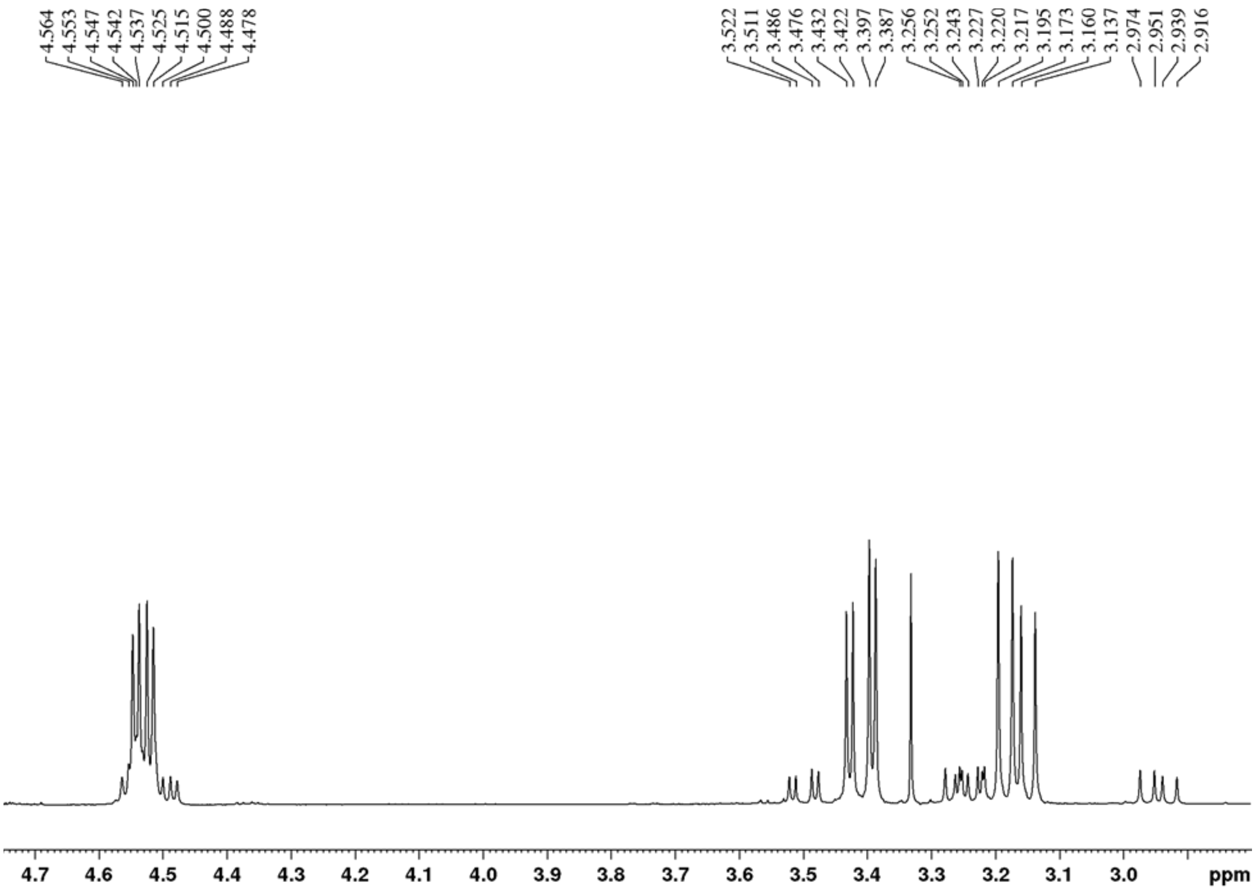

Figure S21. ^{1}H NMR spectrum of NAC trisulfide and elemental sulfur in D_2O at 40 °C for 15 days shows formation of tetrasulfide at (3.50 and 3.25 ppm) and disulfide (3.25 and 2.95 ppm) formation.

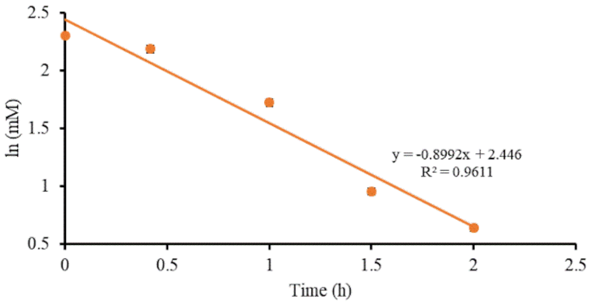

Figure S22. Graph of the degradation of NAC trisulfide under biological conditions in the presence of butylamine fit to pseudo first order reaction kinetics. Due to the equilibrium, only early data points were used. The estimated rate constant was measured to be 0.90 h^{-1}. Error bars are ± instrument error.

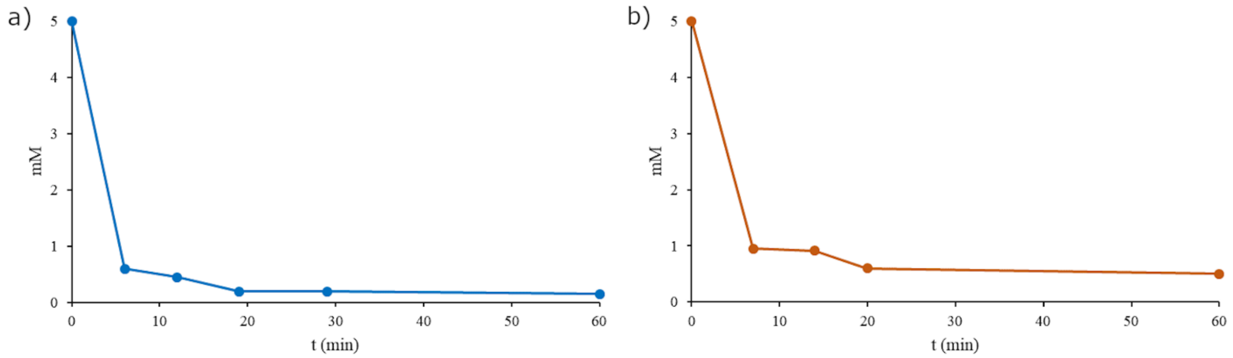

Figure S23. a) Concentration of glutathione trisulfide in the presence of glutathione, and b) concentration of cysteine trisulfide in the presence of cysteine measured by ^{1}H NMR spectroscopy. Each trisulfide degraded very quickly, reaching equilibrium in >15 minutes.
